# Functional analysis of a susceptibility gene (*HIPP27*) in the *Arabidopsis thaliana*-*Meloidogyne incognita* pathosystem by using a genome editing strategy

**DOI:** 10.1186/s12870-023-04401-w

**Published:** 2023-08-11

**Authors:** Tushar K. Dutta, Neeraj Vashisth, Soham Ray, Victor Phani, Viswanathan Chinnusamy, Anil Sirohi

**Affiliations:** 1 Division of Nematology, ICAR-Indian Agricultural Research Institute, New Delhi, 110012 India; 2 Division of Plant Physiology, ICAR-Indian Agricultural Research Institute, New Delhi, 110012 India; 3 Department of Agricultural Entomology, College of Agriculture, Uttar Banga Krishi Viswavidyalaya, Dakshin Dinajpur, Balurghat, West Bengal 733133 India

**Keywords:** Susceptibility factor, CRISPR/Cas9, Overexpression, RT-qPCR, Mutation, Nematode infection

## Abstract

**Background:**

Plant-parasitic root-knot nematodes cause immense yield declines in crop plants that ultimately obviate global food security. They maintain an intimate relationship with their host plants and hijack the host metabolic machinery to their own advantage. The existing resistance breeding strategies utilizing RNAi and resistance (*R)* genes might not be particularly effective. Alternatively, knocking out the susceptibility (*S*) genes in crop plants appears to be a feasible approach, as the induced mutations in *S* genes are likely to be long-lasting and may confer broad-spectrum resistance. This could be facilitated by the use of CRISPR/Cas9-based genome editing technology that precisely edits the gene of interest using customizable guide RNAs (gRNAs) and Cas9 endonuclease.

**Results:**

Initially, we characterized the nematode-responsive *S* gene *HIPP27* from *Arabidopsis thaliana* by generating *HIPP27* overexpression lines, which were inoculated with *Meloidogyne incognita*. Next, two gRNAs (corresponding to the *HIPP27* gene) were artificially synthesized using laboratory protocols, sequentially cloned into a Cas9 editor plasmid, mobilized into *Agrobacterium tumefaciens* strain GV3101, and transformed into Arabidopsis plants using the floral dip method. Apart from 1–3 bp deletions and 1 bp insertions adjacent to the PAM site, a long deletion of approximately 161 bp was documented in the T_0_ generation. Phenotypic analysis of homozygous, ‘transgene-free’ T_2_ plants revealed reduced nematode infection compared to wild-type plants. Additionally, no growth impairment was observed in gene-edited plants.

**Conclusion:**

Our results suggest that the loss of function of *HIPP27* in *A. thaliana* by CRISPR/Cas9-induced mutagenesis can improve host resistance to *M. incognita*.

**Supplementary Information:**

The online version contains supplementary material available at 10.1186/s12870-023-04401-w.

## Introduction

Plant-parasitic nematodes (PPNs) inflict a heavy toll on global crop productivity that may amount to ~ 200 billion US$ annual economic loss [1, 2]. PPNs possess remarkable abilities to alter host development, physiology and immunity. This is facilitated by a ‘toolbox’ of effectors, which is secreted from PPN esophageal glands into the host cell apoplast or symplast during penetration till maintenance of a feeding site in the vascular tissue [3]. The hyper-metabolic feeding cells such as the giant cells (induced by the root-knot nematode *Meloidogyne* spp.) and syncytia (induced by cyst nematodes *Heterodera* and *Globodera* spp.), serve as the permanent nutrient source for the feeding nematodes [4, 5]. Because of the harmful effects of chemical nematicides on the environment and the lack of alternative management strategies, resistance breeding is emphasized as an efficient and sustainable nematode-management strategy. During the last decade, RNAi-based gene silencing strategies (plants expressing double-stranded RNA constructs that target vital nematode genes including effectors) have gained considerable momentum to manage nematode infection in different host plants [6]. However, in the future, targeting nematode effectors is unlikely to be a robust approach because they are already targets of the plant immune system and, therefore, would be under constant selection pressure to diversify and generate resistance-breaking phenotypes [7].

Notably, only a handful of PPN effectors were found to be involved in the zigzag model of plant immune responses by functioning as avirulence (Avr) proteins (SPRYSEC, RBP-1, GrUBCEP12) that interact with plant resistance (R) proteins (Gpa2, Hero, Mi1.2, and Gro1-4) [7–9]. In addition, *R* gene-mediated resistance in most cases is dependent on the recognition of a single pathogen-derived molecular pattern, and pathogens can readily overcome this type of narrow-spectrum resistance [7–9]. Thus, knocking out or mutating the function of a plant susceptibility (*S*) gene that critically facilitates disease compatibility would provide a broader spectrum and durable resistance [10]. To this end, CRISPR/Cas9-based genome editing technology (in which a single guide RNA or sgRNA directs Cas9 endonuclease to create a double-stranded break (DSB) at the targeted DNA site) has been utilized successfully to knock out a number of plant *S* genes (*MLO*, *SWEET* and *eIF4E*), that conferred notable resistance against fungal, bacterial and viral pathogens without incurring in any plant growth impairment [11–13].

However, deployment of CRISPR/Cas9 for achieving nematode resistance in plants is yet an underexplored research area, possibly because a limited number of *S* genes have been reported from the plant-nematode pathosystem. A W-box transcription factor (WRKY45) was found to be a key susceptibility factor that modulated the root-knot nematode (*Meloidogyne javanica*) disease progression in tomatoes [14]. A heavy metal-associated isoprenylated plant protein (HIPP27) was identified as a *S* gene candidate that supported the parasitism of the beet cyst nematode *Heterodera schachtii* in Arabidopsis. The loss of function of *hipp27* in *Arabidopsis* (generated by T-DNA insertional mutagenesis) reduced plant susceptibility to *H. schachtii* infection without affecting the plant phenotype [15]. A spliceosomal protein, SmD1, that interacted with the *M. incognita* effector EFF18, played a crucial role in giant cell ontogenesis, suggesting the probable function of SmD1 as the susceptibility factor in Arabidopsis [16] and tomato [17]. Most recently, pantothenate synthetase (PANC, involved in the vitamin B5 biosynthetic pathway) was identified as an important susceptibility factor for *H. schachtii* infection in Arabidopsis. As nematode *PANC* sequences are quite dissimilar to plant orthologues, nematodes hijack this pathway to meet their nutritional requirements. Loss-of-function mutant lines of Arabidopsis for the *AtPANB1* gene (involved in the penultimate step of the pathway) supported fewer nematodes with reduced fitness, and plant susceptibility to nematodes was restored by overexpressing *AtPANB1* under the control of the 35S promoter in the mutant line [18].

Members of the HIPP family of proteins have different biological functions, such as heavy metal homeostasis and detoxification, transcriptional response to abiotic stresses (drought, cold, salt, heavy metal, abscisic acid, leaf senescence), and plant-pathogen interactions [19–22]. A number of HIPP proteins were shown to act as plant immunity hubs (HIPP26 in *Nicotiana benthamiana* and HIPP19 in *Oryza sativa*), which are targeted by multiple plant pathogens, including potato mop-top virus and the rice blast fungus *Magnaporthe oryzae* [23–25]. *HIPP20* of *O. sativa* was suggested to be a *S* gene that promoted the infection of *M. oryzae* [26]. *HIPP05* of *O. sativa* (alternatively known as *Pi21*) was depicted as a *S* gene, since its loss-of-function mutants conferred durable resistance to *M. oryzae* [27]. In addition, *M. oryzae* hyphal growth was documented in non-host *Arabidopsis*, which was overexpressing the *OsHIPP05* gene, further verifying the role of HIPP05 as a susceptibility factor [28].

HIPPs are the largest metallochaperone family in *A. thaliana* and contain 45 *HIPP* genes, divided into seven major clusters [20]. The zinc-binding *A. thaliana* HIPP3 protein was localized to the cell nucleus, induced after infection with *Pseudomonas syringae* pv. *tomato*, and involved in the regulation of the salicylate-dependent pathogen response pathway, which was antagonistic to the jasmonate pathway [22]. Similarly, zinc-binding *A. thaliana* HIPP26 was localized to the cell nucleus and implicated in various abiotic stress responses [19]. *A. thaliana* HIPP27 was localized to the cell cytoplasm and promoted the infection of *H. schachtii* [15]. To further test the hypothesis that *HIPP27* is a *S* gene, herein, we generated *HIPP27* overexpressor (by using overexpression vectors) and knockout *A. thaliana* lines (by employing the CRISPR/Cas9 system) and investigated the role of *HIPP27* in promoting the infection of the most damaging PPN, *Meloidogyne incognita*. *A. thaliana-M. incognita* is indeed considered as a model pathosystem to study plant-nematode interactions [29].

## Results

### Arabidopsis *HIPP27* sequence is highly conserved in dicotyledonous plants

**Fig. 1:**
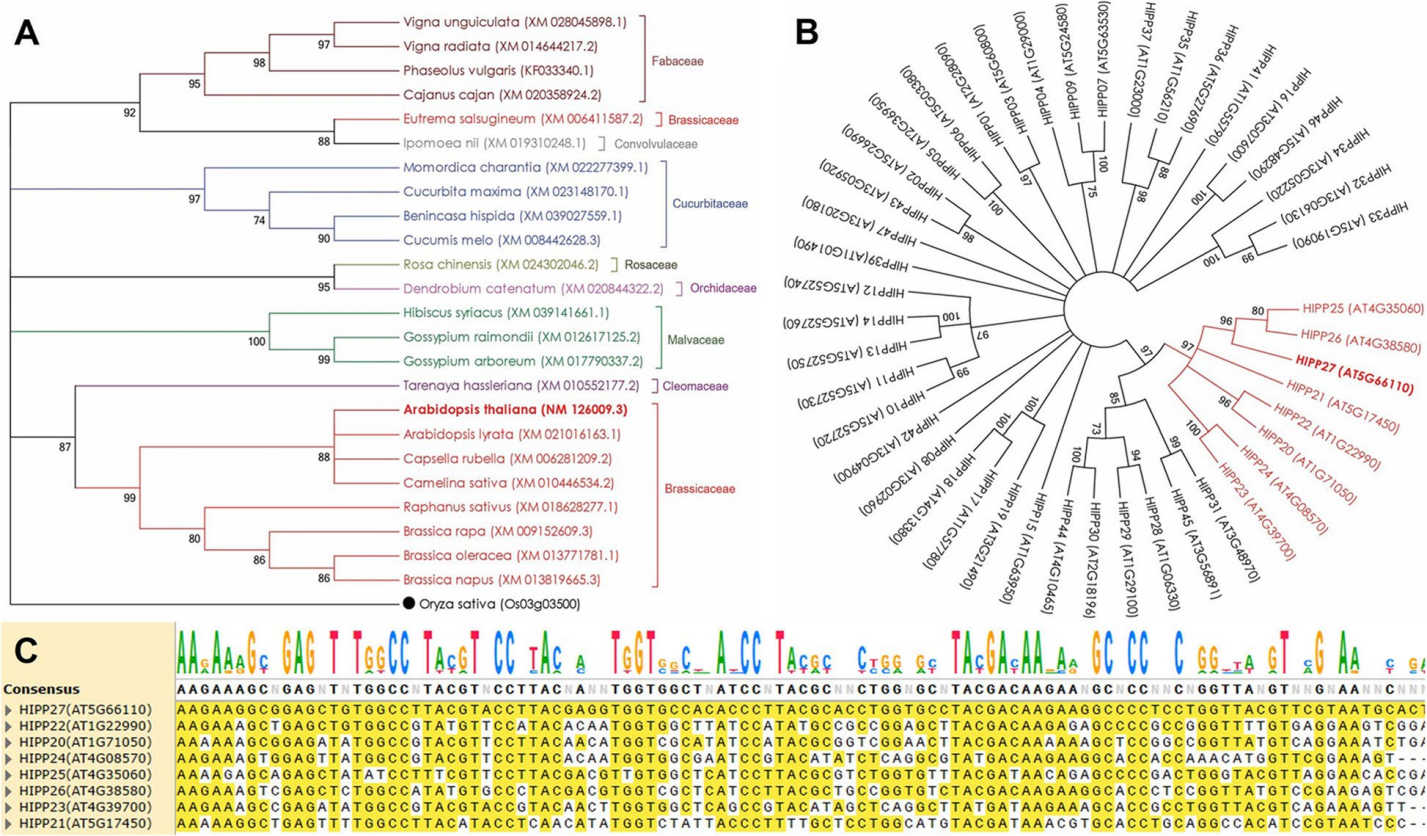
Sequence conservation of the *HIPP27* gene in *Arabidopsis* and other plants. **A** Evolutionary relationships of *A. thaliana hipp27* (entry is indicated in bold font) with their corresponding homologues from other dicot plants. The phylogenetic tree was constructed in MEGA6 software using Maximum Likelihood method based on the Tamura 3-parameter model. Bootstrap consensus was inferred from 1000 replicates, and branches corresponding to less than 70% of replicates were collapsed. The NCBI accession numbers of 25 different entries are provided in parentheses. *HIPP* gene of *O. sativa* was used as the out-group (marked with ●). Entries in different colours represent different plant families, as written beside the clusters. **B** Phylogenetic analysis of different members of the *HIPP* gene family in *A. thaliana*. Gene IDs (retrieved from the TAIR10 genome assembly of *A. thaliana*) for 45 different entries are provided in parentheses. The branch corresponding to closely related homologues of *HIPP27* is indicated in red colour. **C** Multiple sequence alignments indicated discontinuous sequence conservation between closely related homologues of *HIPP27*

The nucleotide sequence of *HIPP27* (Gene ID: AT5G66110) was retrieved from the *A. thaliana* TAIR genome assembly (https://plants.ensembl.org/Arabidopsis_thaliana/) and subjected to BLASTn analysis on the NCBI server using the programme MEGABLAST. Highly similar sequences (Percent identity: 72–100%, Query coverage: 38–100%, E-value: 0) were included in a Maximum Likelihood method-based phylogenetic analysis. The phylogenetic tree was rooted using a *HIPP* sequence from *Oryza sativa* as the out-group. As expected, *HIPP27* from *A. thaliana* formed a distinct clade with *HIPP27* genes from other members (*A. lyrata*, *Capsella rubella*, *Camelina sativa*, *Raphanus sativus*, *Brassica rapa*, *B. oleracea*, *B. napus*) from the Brassicaceae family (Fig. 1A). *HIPP27* from other major dicot families (Malvaceae, Cucurbitaceae, Fabaceae) branched away from the Brassicaceae group. Most importantly, all these members belong to dicot plant families except for an entry from the monocot family Orchidaceae (Fig. 1A). Notably, the amino acid sequence of the *A. thaliana* HIPP27 protein showed a high degree of conservation with HIPP27 proteins of different cotton genotypes, including *Gossypium hirsutum* (Percent identity: 70.75%), *G. arboreum* (72.79%), and *G. raimondii* (72.11%) (Supplementary Figure S1).

In order to find the highly homologous sequences of *HIPP27* in *A. thaliana*, sequence conservation of *HIPP27* across the known 45 *HIPP* sequences of *A. thaliana* was studied. A phylogenetic tree showed a distinct clade of HIPP27 that contained *HIPP25* (nucleotide sequence identity with *HIPP27* – 61.45%), *HIPP26* (67.80%), *HIPP21* (59.05%), *HIPP22* (53.99%), *HIPP20* (56.34%), *HIPP24* (53.43%) and *HIPP23* (54.63%) (Fig. 1B). Multiple sequence alignments of *HIPP* sequences (a highly homologous stretch is shown here) showed discontinuous sequence conservation (Fig. 1C), suggesting that *HIPP27* is a unique gene in the *A. thaliana* genome.

In order to analyze the transcription pattern of *HIPP27* in different developmental stages and plant parts of *A. thaliana*, RT-qPCR analysis was performed. No significant change (*P* > 0.05) in *HIPP27* expression in different plant parts (roots, shoots, leaves, flowers, and seeds) and developmental stages (7, 14, 21, and 30 days) was observed (Supplementary Figure S2), indicating that *HIPP27* is ubiquitously expressed in *A. thaliana*.

### *HIPP27* overexpression increased *A. thaliana* susceptibility to *M. incognita*

**Fig. 2:**
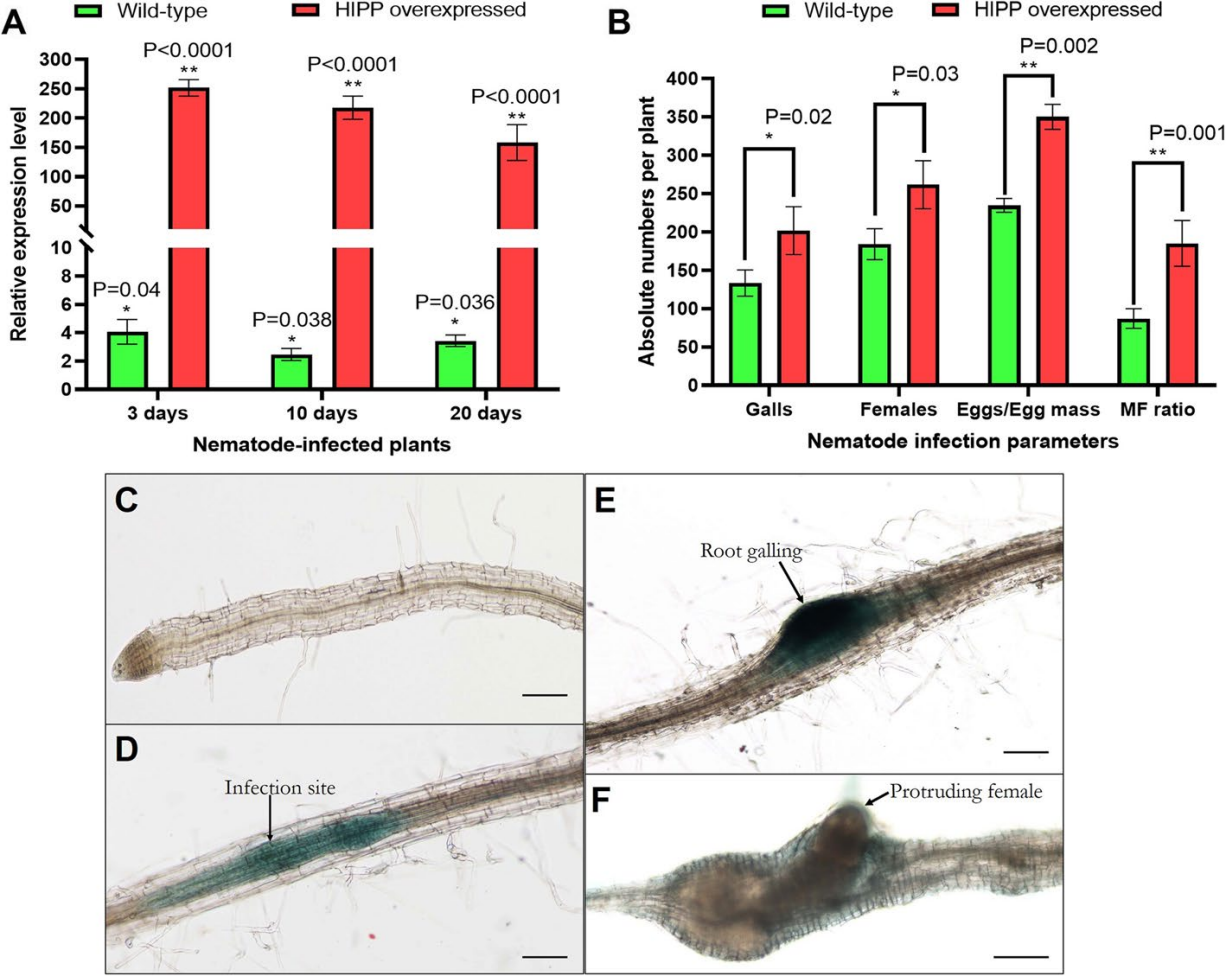
Overexpression of *HIPP27* increases the susceptibility of *A. thaliana* to *M. incognita*. **A** qPCR validation of an increase in *HIPP27* transcript level (RNA was extracted from the root tissues) in an overexpression line upon *M. incognita* infection at 3, 10, and 20 days. Asterisks (*P < 0.05, **P < 0.01; paired t-test) indicate significant differential expression of *HIPP27* mRNA in wild-type and overexpression plants compared to its baseline expression in non-infected control plants (fold change values were set at 1). Gene expression was normalized using two housekeeping genes of *A. thaliana* (ubiquitin and *18S rRNA*). Each bar represents the mean fold change value ± standard error (SE) of qPCR runs in three biological and technical replicates. **B** The average number of galls, females, eggs per egg mass, and multiplication factor (MF) ratio per root system were increased in the overexpression line compared to the wild-type at 30 days after infection. Bars represent the mean ± SE (*n* = 10). Asterisks indicate a significant difference between two treatments (*P < 0.05, **P < 0.01; Tukey’s HSD test). Photomicrographs depict the expression of *HIPP27* promoter:*GUS* fusion in *A. thaliana* root upon *M. incognita* infection. **C** Two-week-old root at 0 days post infection (dpi). *M. incognita*-infected root at 3 (**D**), 10 (**E**), and 20 (**F**) dpi. Scale bar = 100 μm

Using zero background TA-cloning vectors, we generated the *HIPP27* overexpression lines in the *A. thaliana* Col-0 background (under the control of a constitutive promoter). Progeny plants of a homozygous line were inoculated with 500 *M. incognita* second-stage juveniles (J2s). At 3, 10, and 20 days post infection (dpi), approximately a 251-, 217-, and 158-fold increase (*P* < 0.0001) in *HIPP27* transcripts was documented in overexpression plants, respectively, compared to control plants (Fig. 2A). The average number of galls, females, eggs per egg mass, and nematode multiplication factor (MF) ratio were significantly increased (*P* < 0.05) in overexpression plants compared to wild-type plants at 30 dpi (Fig. 2B), suggesting that increased *HIPP27* expression might have caused increased plant susceptibility to nematodes.

To further investigate whether *HIPP27* expression is correlated with nematode infection, *A. thaliana* Col-0 plants were transformed with a *pHIPP27*:*GUS* construct that contained the promoter region of *HIPP27* (1 Kb upstream of the start codon) fused with the β-glucuronidase reporter gene. Progeny plants from a homozygous line were inoculated with *M. incognita* J2s. Roots were stained for GUS activity at different time points. No staining was detected in the root apex and elongation zone of the primary root (Fig. 2C). A strong GUS expression was detected at the nematode infection site (presumably the site of giant cell induction) at 3 dpi (Fig. 2D) and the staining became more intense at 10 dpi with root swelling at the infected site (Fig. 2E). However, GUS staining was weakly detected at 20 dpi in the galled roots (Fig. 2F), suggesting that *HIPP27* expression is particularly induced in *A. thaliana* roots during the early infection stage of *M. incognita*.

### CRISPR/Cas9-targeted mutagenesis of *Athipp27* and generation of ‘transgene-free’ mutants

**Fig. 3:**
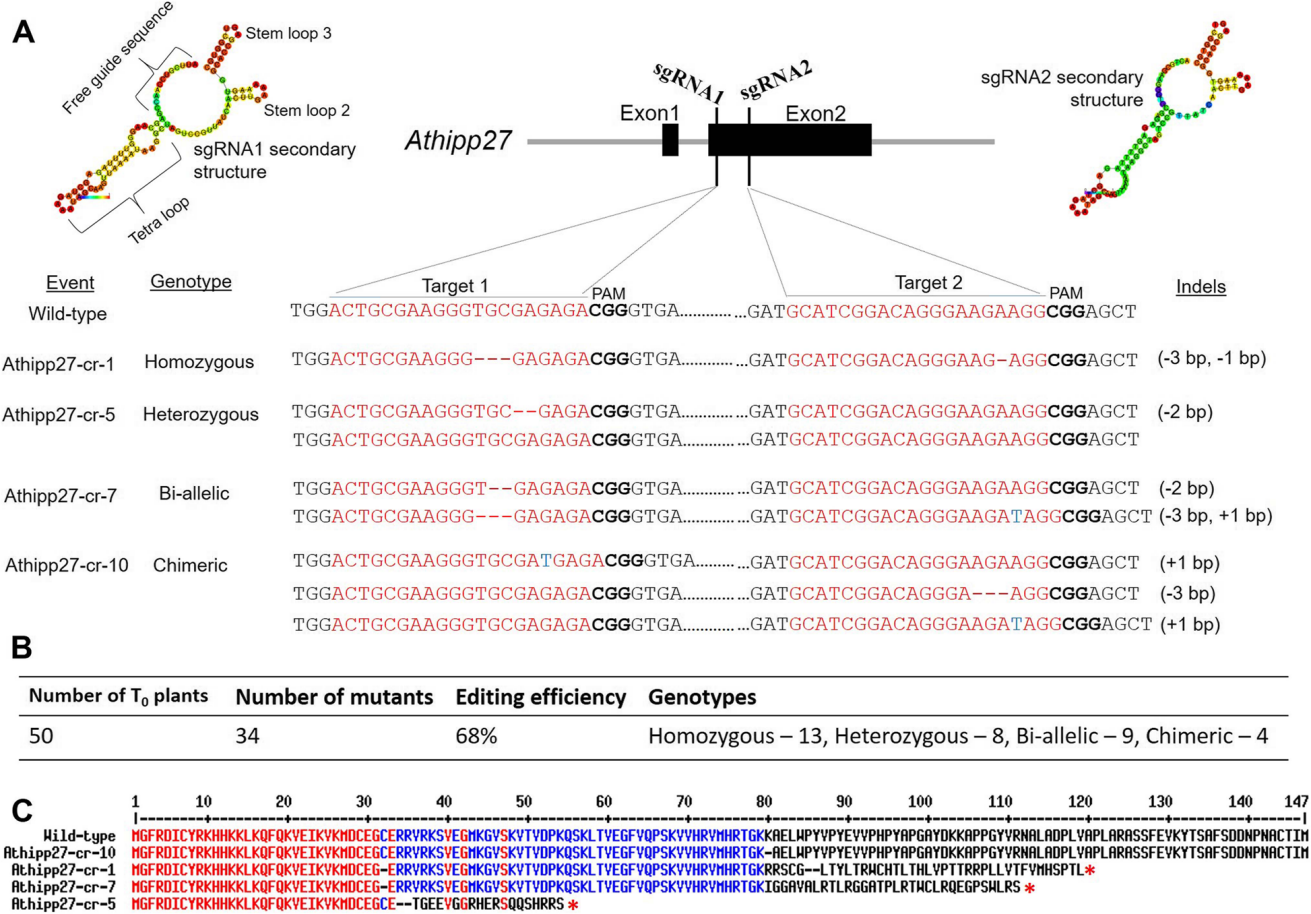
CRISPR/Cas9-targeted mutagenesis of the *Athipp27* gene in *A. thaliana*. **A** Mutant identification in T_0_ plants. sgRNA-targeting sites were located on exon 2 of the *Athipp27* gene. Predicted secondary structures of sgRNAs indicate ideal folding of gRNA scaffolds for efficient editing. Based on sequencing results, four different types of mutation (homozygous, heterozygous, bi-allelic and chimeric) were detected in different T_0_ events. Red hyphens and blue letters denote deletions and insertions, respectively. **B** Summarization of the editing efficiency and genotype of different T_0_ plants. **C** Alignment of the deduced amino acid sequences of the *Athipp27* gene (codes for 147 aa) in wild-type and different mutant events. Asterisks indicate translation termination

For targeted disruption of *Athipp27* by CRISPR/Cas9, two sgRNAs targeting the second exon of *Athipp27* were designed using different in silico tools. sgRNAs were targeted from the conserved regions of AT5G66110 transcript and its three transcript variants (AT5G66110.1, AT5G66110.2 and AT5G66110.3) in order to ensure knockout probabilities in all the splice variants of *Athipp27* gene. The two sgRNAs and their protospacer adjacent motif (PAM) sequences are shown in Fig. 3A. The efficient selection of sgRNAs were initially validated by secondary structure prediction. Both the sgRNA structures contained the maximum free guide sequence (minimal internal base pairing in the guide sequence leads to greater target recognition), an intact tetra loop (repeat crRNA and anti-repeat tracrRNA), stem loop 2 and 3 (in tracrRNA) (Fig. 3A). The intact stem loop structures promote stable Cas9-sgRNA-DNA complex formation and improve in vivo editing efficacy. An edit vector pHEE401:Athipp27 expressing *Cas*9, two gRNAs, their scaffolds, and hygromycin antibiotic resistance gene were constructed, mobilized into *Agrobacterium tumefaciens* strain GV3101, and transformed into *A. thaliana* Col-0 via the floral dip method.

Twelve independent T_0_ transgenic events were obtained that were designated as Athipp27-cr-1 to Athipp27-cr-12. Sequencing-based genotyping showed that four different types of insertion-deletion (indel) mutations, i.e. homozygous, heterozygous, bi-allelic and chimeric, were detected in the progeny plants of different events (Fig. 3A). Overall, an editing efficiency of 68% was recorded in our study (Fig. 3B). Amino acid sequence analysis of the mutant events showed premature translation termination of HIPP proteins (Fig. 3C), suggesting the probable disruption of gene function. Notably, very high out-of-frame scores for targets 1 (73.55) and 2 (68.68) were recorded while sgRNA designing was performed.

**Fig. 4:**
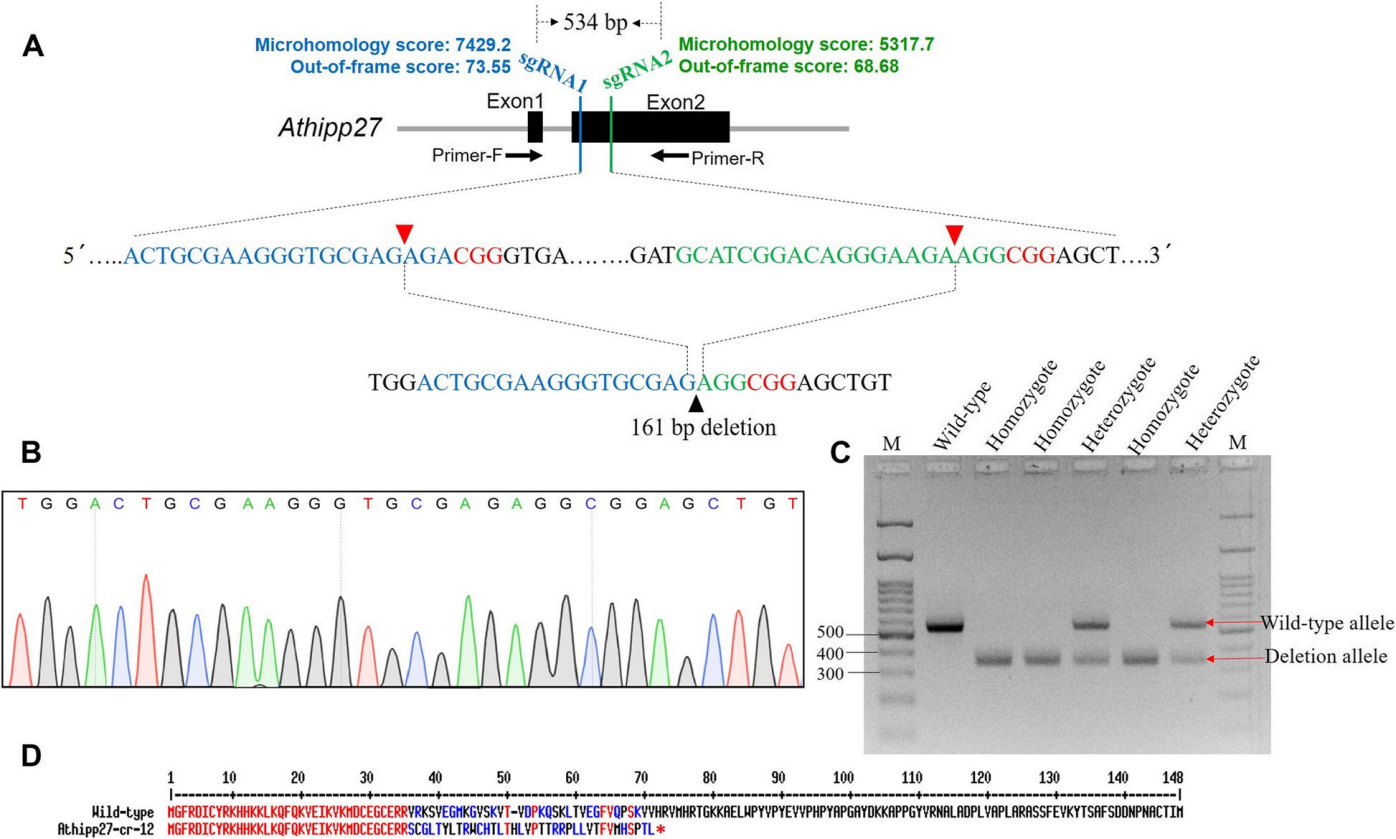
CRISPR/Cas9-induced large deletion of the *Athipp27* gene in *A. thaliana*. **A** Schematic diagram of the sgRNA-targeting sites. sgRNA 1 and 2 sequences are indicated in blue and green, respectively. The PAM site is indicated in red colour. The Cas9 cleavage site is indicated by a red triangle. The use of two sgRNAs led to the deletion of a larger DNA fragment of 161 bp in the mutant T_0_ event Athipp27-cr-12. **B** A representative chromatogram of the DNA sequence of a mutant homozygote is given. **C** Agarose gel electrophoresis showing both homozygous (only deletion allele) and heterozygous (both deletion and wild-type allele) mutants in different clonal lines of Athipp27-cr-12. The DNA fragments flanking the two sgRNA sites were amplified by PCR and were 534 bp in the wild-type and 373 bp in the mutant. M – 100 bp DNA ladder. **D** Alignment of the deduced amino acid sequences of the *Athipp27* gene in wild-type and event Athipp27-cr-12. Asterisk indicates translation termination

A large deletion of 161 bp in the target gene was also found in event Athipp27-cr-12 (Fig. 4). Apart from sequencing (Fig. 4B), deletion alleles (homozygous and heterozygous) were detected in different individual plants by PCR using primers that flanked the two target sites. DNA fragments flanking the two target sites were 534 bp in the wild-type and 373 bp in the mutant (Fig. 4C). Amino acid sequence analysis of the mutant event showed premature translation termination of the HIPP protein (Fig. 4D). We hypothesize that the large fragment deletion in this case is the resultant effect of the microhomology-mediated end joining (MMEJ) repair mechanism. A very high microhomology score for targets 1 (7429.2) and 2 (5317.7) was recorded while sgRNA designing was performed (Fig. 4A). Our qPCR analysis showed that *HIPP27* expression was significantly attenuated in the edited line Athipp27-cr-12 compared to the wild-type (*P* < 0.01), whereas expression of other closely-related genes, including *HIPP20*, *HIPP21*, *HIPP22*, *HIPP23*, *HIPP24*, *HIPP25*, and *HIPP26*, was unaltered in the edited and wild-type plants (*P* > 0.01; Supplementary Figure S3).

Probable off-target sites of the two sgRNAs were predicted using Cas-OFFinder and CRISPR-P 2.0 web tools. Genomic DNA was isolated from T_0_ generation *A. thaliana* plants, subjected to PCR (using off-target site-specific primers) and sequencing. No induced mutations were detected in the off-target loci of mutant plants (Table 1).

**Table 1:** Analysis of the putative off-target effect at potential off-target sites of the *Arabidopsisthaliana* mutants. Mismatching bases are indicated in bold. Nucleotides in italics are PAM sequences. Off-target loci located in the intergenic region were not sequenced

**gRNA**	**Putative off-target locus**	**Off-target sequence**	**Number of mismatches**	**Number of T**_**0**_ **plants sequenced**	**Number of plants with mutations**
Target 1	Chr2:+4859863	A**G**TGCGAAGGGT**TT**GAGAGA*CGG*	3	Intergenic region	
	Chr3:-12975949	ACTG**TT**AAGG**C**TGCGAGAGA*AGG*	3	Intergenic region	
Target 2	Chr1:+5566223	**C**CATC**T**GACA**T**GGAAGA**T**GG*TGG*	4	5	0
	Chr1:+22430091	GCAT**G**GGA**GT**GGGAAGAA**A**G*AGG*	4	5	0
	Chr4:-17280777	GCATC**T**G**C**CA**C**GGA**G**GAAGG*TGG*	4	5	0

**Fig. 5:**
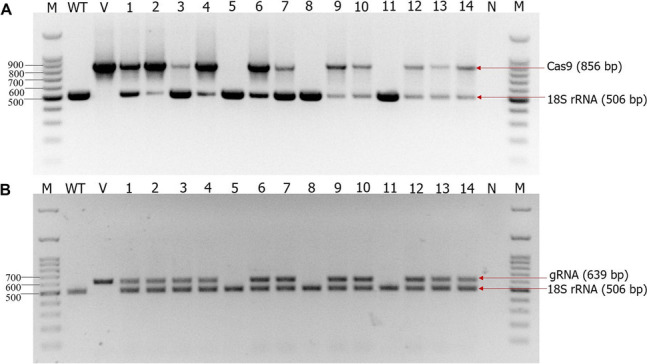
Detection of ‘transgene-free’ homozygous *Athipp27* knockout mutants in the T_1_ generation. **A** Detection of a partial Cas9 coding sequence (856 bp) in different mutant individuals. **B** Detection of sgRNA (639 bp), a region spanning from the sgRNA scaffold to downstream vector in different mutant individuals. *A. thaliana 18S rRNA* gene (506 bp) was used as the reference in multiplex PCR reactions. M – 100 bp DNA ladder, WT – wild-type plants, V – plasmid vector, N – negative control without template DNA

To identify ‘transgene-free’ homozygous *Athipp27* knockout mutants, the presence or absence of *Cas9* and sgRNA in transgenic *A. thaliana* plants of T_1_ generation (obtained via self-pollination of T_0_ plants) was determined by multiplex PCR (primers specific to a housekeeping gene, *18S rRNA*, were included in the reaction mixture as a reference). Three mutant plants (plant numbers 5, 8, and 11) inherited from the Athipp27-cr-12 line did not generate *Cas9*- and sgRNA-specific amplicons (Fig. 5A, B). As these mutants did not contain any transgenic elements of *hipp27*-sgRNA/Cas9 vectors, we considered them as ‘transgene-free’ homozygous mutants.

### Loss of function of HIPP27 increased *A. thaliana* resistance to *M. incognita* without impairing the host basal defense

**Fig. 6:**
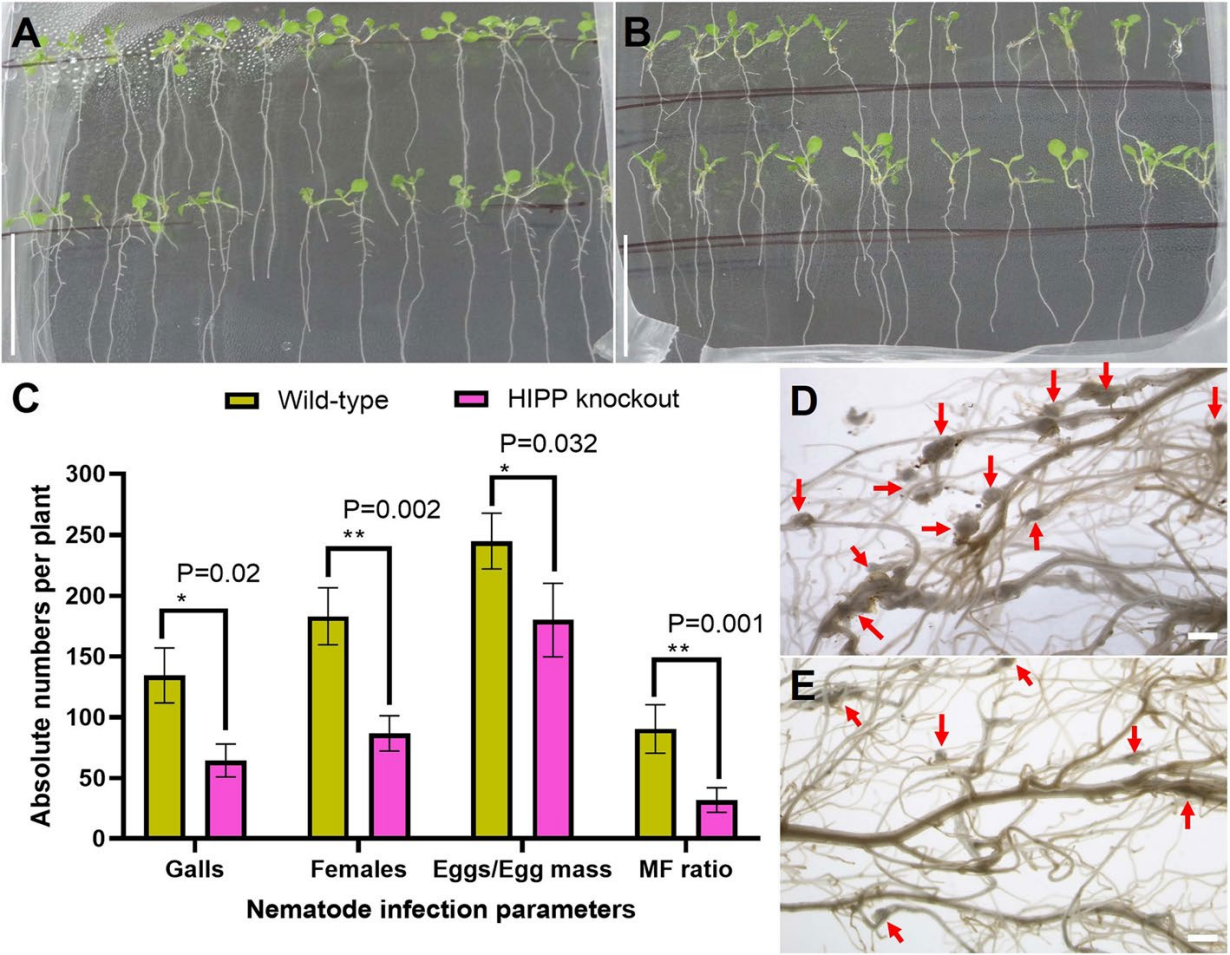
The loss of function of HIPP27 reduced *A. thaliana* susceptibility to *M. incognita*. Growth phenotypes of wild-type (**A**) and ‘transgene-free’ *Athipp27* knockout mutants (**B**) showed no adverse effect on plant shoot and root morphology due to the induced mutation in the *hipp27* gene. Scale bar = 3 cm. **C** The average number of galls, females, eggs per egg mass, and multiplication factor (MF) ratio per root system decreased in mutants compared to the wild-type at 30 days after infection. Bars represent the mean ± SE (*n* = 10). Asterisks indicate a significant difference between two treatments (*P < 0.05, **P < 0.01; Tukey’s HSD test). Photomicrographs demonstrate greater galling intensity in wild-type roots (**D**) compared to mutants (**E**). Scale bar = 500 μm

By further self-pollinating ‘transgene-free’ homozygous Athipp27-cr-12 plants, a T_2_ generation was obtained that was subjected to growth phenotype analysis and ‘challenge inoculation’ with nematodes. No adverse effect on plant shoot and root morphology was documented due to the induced mutation in the *hipp27* gene according to the comparative growth phenotypes of wild-type and mutant plants at 14 days after germination (Fig. 6A, B). No significant difference in average dry weight, root length, flowering time and plant height between wild-type and mutant plants was observed (*P* > 0.05; Supplementary Figure S4).

Each T_2_ plant was infected with 500 J2s of *M. incognita* in a pot, and plants were harvested at 30 dpi to measure different susceptibility parameters. Compared to wild-type plants, mutant plants exhibited significantly lower susceptibility to *M. incognita* in terms of average number of galls (Percent reduction – 52.11%, *P* = 0.02), females (52.64%, *P* = 0.002), eggs per egg mass (26.53%, *P* = 0.032) and MF ratio (64.87%, *P* = 0.001) (Fig. 6C). As expected, the galling intensity was comparatively greater in wild-type roots compared to mutant ones (Fig. 6D, E). Overall, these results suggest that *A. thaliana HIPP27* plays a crucial role in *M. incognita* parasitism.

To examine whether the reduced susceptibility of mutant plants to *M. incognita* is a result of altered plant defense responses, we carried out RT-qPCR analysis to quantify the expression of selective *A. thaliana* basal defense marker genes in the shoots and roots of nematode-infected wild-type and gene-edited plants at 2 days after inoculation. The marker genes include peroxidase, *MPK4* (related to oxidative stress), *EDS1*, *PAD4*, *PR1*, *PR2* (salicylic acid pathway), *PDF1.2*, *HEL1* (jasmonic acid pathway), *ERF6* and *ACS2* (ethylene signaling). The transcript abundance of all 10 marker genes did not differ significantly between wild-type and edited plants (*P* > 0.05; Supplementary Figure S5), indicating the specific role of *HIPP27* in conferring nematode susceptibility.

## Discussion

Arabidopsis *HIPP27* was previously shown to act as a *S* gene that facilitated the infection of the beet cyst nematode *H. schachtii* in *A. thaliana*; loss-of-function mutants were generated by T-DNA insertional mutagenesis [15]. The present study describes the role of *HIPP27* as an important susceptibility factor for southern root-knot nematode *M. incognita* infection in *A. thaliana*. We generated the loss-of-function mutants using a CRISPR/Cas9-mediated targeted mutagenesis approach. To date, CRISPR/Cas9-based editing has been utilized to investigate the molecular basis of plant–nematode interactions rather than conducting research on *S* genes and creating mutants to achieve PPN resistance [30–32]. In a recent report, CRISPR/Cas9-based knockout of the *S* gene, *OsHPP04*, conferred rice (cv. Nipponbare) resistance to the root-knot nematode *M. graminicola* [33]. Our study provides further insights into the CRISPR/Cas9-mediated functional analysis of nematode-responsive *S* genes in plants.

Dong et al. [30] utilized the CRISPR/Cas9 system to knock out the syntaxin gene of the t-SNARE family in the nematode‐resistant soybean cultivar Peking. Intriguingly, Peking roots with mutations in the syntaxin gene exhibited increased susceptibility to *H. glycines* infection, suggesting the critical role of t-SNAREs in conferring *H. glycines* resistance. Using CRISPR/Cas9, a putative *R* gene (*MG1*) was functionally validated in rice. Compared to wild type, homozygous mutant lines of ZH11 (japonica variety), SL 22–620 and HKG 98 (aus rice) conferred susceptibility to *M. graminicola*, indicating that *MG1* is required for *M. graminicola* resistance [31]. Similarly, CRISPR/Cas9-induced mutagenesis in the *OsPR10/OsBet v1* gene conferred greater susceptibility of rice to *M. graminicola*, suggesting that *OsBet v1* is involved in rice defense against nematodes [32]. A CRISPR/Cas9-induced mutation in the *WRKY45* gene exhibited substantially lower *M. incognita* infection in tomatoes, thus revealing that SlWRKY45 may act as a negative regulator of tomato defense against *M. incognita* [34].

Arabidopsis HIPP proteins are the largest family of metallochaperones that contain a heavy metal-binding (HMA) domain [20]. A number of plant immune receptors possess HMA domains, implicating their putative role in host defense against pathogens [35]. Different HIPP orthologues, including HIPP26 in *N. benthamiana* and HIPP19 in *O. sativa*, act as plant immune response modulators that target multiple pathogen effectors [23–25]. *HIPP05* and *HIPP20* of *O. sativa* were reported as *S* genes that facilitated *M. oryzae* proliferation in the host tissue [26–28]. *A. thaliana* HIPP3 was localized to the cell nucleus, and was found to regulate the salicylate-dependent pathway of pathogen response via binding zinc [22]. Similarly, *A. thaliana* HIPP26 was localized to the cell nucleus, and by binding to a zinc finger transcription factor, it was found to regulate the drought stress response [19]. On the contrary, *A. thaliana* HIPP27 was localized to the cell cytoplasm only [15]. In this study, we showed that *A. thaliana HIPP27* is ubiquitously expressed in different plant parts and across different developmental stages. Phylogenetic analysis indicated that *HIPP27* is highly conserved across the dicotyledonous plants and most specifically in the Brassicaceae family. However, *HIPP27* was found to be a unique member of the metallochaperone family when its sequence was compared with other *HIPP* genes from *A. thaliana*.

In the present study, we generated HIPP overexpression lines to assess whether increased transcription of *HIPP27* conferred greater susceptibility of *A. thaliana* to *M. incognita*. As expected, the average number of galls, females, eggs per egg mass, and MF ratio (which determines nematode reproductive success in the host plant; [36]) were greatly increased in overexpression plants compared to the wild-type. In addition, *HIPP27* fusion with a GUS reporter construct demonstrated that nematode infection potential is very much correlated with *HIPP27* expression. A similar GUS assay was conducted to identify the nematode-responsive promoter elements in *A. thaliana* [37, 38].

Next, we generated a number of genome-edited lines that harboured the different knockout variants of the *hipp27* gene. We designed two sgRNAs from the second exon of *Athipp27* (as the first exon was quite smaller in size and did not contain conserved regions across the *hipp27* transcript variants). The secondary structure of each sgRNA (which interacts with the Cas9 protein in vivo and thus improves editing efficiency) contained a fairly greater stretch of free guide sequence and intact stem loop structures, which was indicative of greater stability in the sgRNA structure [39]. We encountered a large deletion of 161 bp in an event called Athipp27-cr-12, probably because of the MMEJ-mediated DSB repair mechanism. We recorded greater microhomology scores for both sgRNAs, suggesting the proclivity of DSBs (at two target sites) to undergo the MMEJ repair pathway. Notably, a deletion of 248 bp was encountered in one of the transgenic rice events when the *MG1* gene was knocked out via CRISPR/Cas9 [31]. Apart from non-homologous end joining (NHEJ) and homology-directed repair (HDR), the MMEJ repair mechanism has emerged as a major DSB repair pathway in plant genome editing applications [40].

We did not find any induced mutations in the off-target loci of the transgenic plants. Event number Athipp27-cr-12 was taken forward (as premature translational stoppage was documented in the HIPP amino acid sequences, presumably no functional HIPP protein was produced) for generating homozygous T_2_ mutants. Transgene-free mutants were ‘challenge inoculated’ with *M. incognita* J2s in the controlled environment. At 30 dpi, 52.11, 52.64, 26.53, and 64.87% reductions in gall number, nematode females, eggs per egg mass, and MF ratio, respectively, were documented in mutants compared to wild-type plants. Additionally, mutant plants did not show any pleiotropic effect on plant phenotypes. The expression of defense response genes did not significantly differ between wild-type and mutant plants, indicating that the reduced susceptibility of HIPP mutants to *M. incognita* is directly related with the loss of function of *HIPP27* gene. Any possibility of CRISPR-induced positive or negative effect on plant defense gene expression can be excluded in our study. Notably, the loss of function (via T-DNA mutation) of *HIPP27* did not impair plant basal defense in *A. thaliana* shoots treated with flg22 immunopeptide [15].

In conclusion, our CRISPR-Cas9-induced targeted mutagenesis of the *HIPP27* gene established *Athipp27* as a susceptibility factor for *M. incognita* infection in *A. thaliana*. As *S* genes are recessively inherited in host plants, induced mutations in *S* genes are likely to be durable and may confer broad-spectrum resistance [10]. The findings of this study can facilitate our understanding of the role of *S* genes in other plant-nematode pathosystems, and similar *S* genes may be targeted in crop plants (such as cotton) to generate nematode-resistant genome-edited lines.

## Materials and methods

### Culturing of nematodes

A pure culture of *M. incognita* race 1 was propagated in the roots of eggplant (*Solanum melongena* cv. Pusa Purple Long) in pots in a growth chamber at 28˚C with 16: 8 h light: dark photoperiod (light level – 300 μmol m^−2^ s^−1^). At 2 months after nematode inoculation, plants were harvested, egg masses were handpicked from the infected roots and hatched in sterile tap water at 28ºC. Freshly hatched J2s were used for all the experiments.

### Plant growth conditions and nematode infection

Seeds of *Arabidopsis thaliana* ecotype Columbia-0 (Col-0) were surface sterilized with 70% ethanol (for 2 min), 0.1% HgCl_2_ and 0.1% SDS (for 5 min), followed by rinsing in sterile distilled water. Seeds were germinated in Petri plates containing the MS media. Plates were incubated at 21ºC with 16: 8 h light: dark photoperiod. Two-week-old seedlings were transferred to 500 ml pots containing soil rite (Keltech Energies Ltd., Bengaluru). Plants were grown in the growth chamber (at the National Phytotron Facility, ICAR-IARI) at 21ºC with 16: 8 h light: dark photoperiod. After 3 weeks, each plant was inoculated with 500 M*. incognita* J2s near the root zone using a sterilized pipette tip. Plants were harvested at different time points to examine different nematode infection parameters, i.e., number of galls, females (or egg mass), eggs per egg mass, and multiplication factor [(number of egg mass × number of eggs per egg mass) ÷ initial inoculum level]. An identical procedure was followed for analyzing the nematode infection level in transformed plants. At least 10 plants were included in each treatment, and the whole experiment was repeated at least three times.

### Bioinformatics analyses

Sequences of different candidate genes were exported from the NCBI database and the *A. thaliana* TAIR genome assembly. Exon, intron, coding sequence, and 5´ and 3´ UTR in the sequence were analyzed in the FGENESH (https://www.softberry.com/) and Expasy (https://web.expasy.org/) webservers. Conserved domains and motif signatures were assessed in the NCBI conserved domain database (https://www.ncbi.nlm.nih.gov/Structure/cdd/) and the InterProScan database (https://www.ebi.ac.uk/interpro/). Sequences were aligned with their homologues from different plant species via the Clustal Omega tool. A phylogenetic tree was constructed using the MEGA6 tool by following the Maximum Likelihood method based on the Tamura 3-parameter model. The tree was generated with the highest log likelihood (-15,493.9900). A discrete Gamma distribution was used to model evolutionary rate differences among sites (5 categories (+ *G*, parameter = 1.5549)).

### Generation of *Athipp27*-overexpression plants

pCXSN (a zero background TA cloning-based vector; [41]) was digested with *Xcm*I (New England Biolabs) to create T-overhangs. In parallel, total RNA was extracted from the two-week-old *Arabidopsis* plants by using the NucleoSpin RNA Plant Kit (TaKaRa) in accordance with the manufacturer’s instructions and reverse-transcribed to cDNA by using the SuperScript VILO cDNA synthesis Kit (Invitrogen). The coding sequence of *Athipp27* (444 bp) was PCR-amplified (primer detail is provided in Supplementary Table S1) by using proofread-capable Phusion DNA polymerase (Invitrogen) and A nucleotide overhang were created in PCR products by following the A-tailing protocol (https://www.promegaconnections.com/a-quick-method-for-a-tailing-pcr-products/). Gel-purified digested pCXSN vector and *Athipp27* PCR product were ligated via T4 DNA ligase (Invitrogen) with standard vector to insert molar ratio of 1:3 [42]. pCXSN: *Athipp27* was initially transformed into *E. coli* DH5α cells by electroporation, sequence verified, and subsequently transformed into *A. tumefaciens* strain GV3101 via the freeze–thaw method. The overexpression vector contained the CaMV 35S promoter.

Genomic DNA was extracted from two-week-old Arabidopsis plants using the NucleoSpin Plant II Kit (TaKaRa) in accordance with the manufacturer’s instructions. The promoter region of *Athipp27* (1 Kb upstream of the start codon) was PCR-amplified (primer detail provided in Supplementary Table S1) from the genomic DNA and ligated into pCXGUS-P [41] by using the TA cloning method as described above. pCX-GUS-P: *Athipp27* was transformed into *A. tumefaciens* strain GV3101 as described above.

Four- to six-week-old *A. thaliana* Col-0 wild-type plants (germinated in Petri plates containing MS media followed by transfer to pots containing soil rite) were transformed with *A. tumefaciens* by the floral dip method [43]. After drying the plants, T_0_ seeds were harvested and sterilized before being screened on MS media plates containing the antibiotic hygromycin (25 mg L^−1^). Transformants (3–4 leaf stage) that survived in antibiotics were transferred to pots containing soil rite in the growth chamber (Supplementary Figure S6). T_3_ homozygous plants were used for infection experiments.

### GUS staining

Histochemical validation of GUS activity was analyzed using 5-bromo-4-chloro-3-indolyl-b-D glucuronide (XGluc) as the substrate. Nematode-infected roots at different stages (3, 10, and 20 days after infection) were carefully washed free of soil and submerged in a freshly-prepared GUS staining solution (0.5 mM X-Gluc, 0.1 M NaHPO_4_, 0.5 mM K_3_Fe(CN)_6_, 0.5 mM K_4_Fe(CN)_6_, 0.01 M EDTA, 20% methanol, 0.1% Triton X-100) for overnight at 37ºC. Root tissues were cleared by replacing the solution with 70% alcohol, and photomicrographs were taken with a Zeiss Axiocam MRm microscope.

### Gene expression analysis

Total RNA was extracted from nematode-infected plants as described above. The integrity of the RNA molecule was checked by electrophoresis in a 1% (w/v) agarose gel. RNA purity and quantity were examined in a Nanodrop spectrophotometer (Thermo Fisher Scientific). Approximately 1 µg of RNA was reverse-transcribed to cDNA using the SuperScript VILO cDNA synthesis Kit (Invitrogen). A qPCR-based transcriptional profile of different candidate genes (primer details are provided in Supplementary Table S1) was carried out in a CFX96 thermal cycler (BioRad). The qPCR reaction volume (10 μL) contained 1.5 ng cDNA, 750 nM each of forward and reverse primers, and 5 μL SYBR Green PCR master-mix (BioRad). qPCR amplification conditions were as follows: a hot start phase of 95 °C for 30 s, 40 cycles of 95 °C for 10 s and 60 °C for 30 s. In addition, a melt curve programme (95 °C for 15 s, 60 °C for 15 s, then a slow ramp from 60 to 95 °C) was followed to visualize the qPCR amplification specificity. Quantification cycle (Cq) values were exported from CFX Maestro software (BioRad). Arabidopsis housekeeping genes (*18S rRNA* and ubiquitin) were used as internal references for normalizing the candidate gene expression data. The fold change in the target gene was calculated using the 2^−ΔΔCq^ method. The qPCR run contained three biological and three technical replicates for each sample.

### Designing of guide RNAs

Using the *Athipp27* sequence as the query, gRNAs were designed via different in silico tools (RGEN—https://www.rgenome.net/; CHOPCHOP—https://chopchop.cbu.uib.no/; CRISPick—https://portals.broadinstitute.org/; MMEJ-KO—http://skl.scau.edu.cn/mmejko/). Based on greater microhomology predictor (repairs two DSBs using microhomology-mediated end joining (MMEJ)), out-of-frame score (ensures codon shifting), minimal self-complementarity, minimal off-target (by using Cas-OFFinder tool—http://www.rgenome.net/cas-offinder/) effect, and secondary structure prediction (RNAfold webserver (http://rna.tbi.univie.ac.at/cgi-bin/RNAWebSuite/RNAfold.cgi) predicts stem-loop and hairpin formation in the intended crRNA: tracrRNA hybrids), gRNAs were shortlisted.

### Generation of CRISPR/Cas9 constructs

In order to assemble two gRNAs, two gRNA spacer or target sequences (20 bp each) along with the *Bsa*I recognition site were incorporated into PCR forward and reverse primers, respectively. Four primers were used that contained overlapping sequences. Using the pCBC vector (Addgene) as a template, a single PCR fragment was amplified that contained target 1, gRNA scaffold, *Arabidopsis* U6 terminator, promoter, and target 2. Next, the purified PCR fragment was assembled into the multiple cloning site of the Cas9-expressing binary vector pHEE401 (Addgene) by using the Golden Gate cloning method [44, 45]. The recombinant pHEE401 (pHEE401:Athipp27-cr) contained two gRNA expression cassettes driven by U6 promoters and terminators and codon-optimized Cas9 expressed under the control of an egg cell-specific promoter and *rbcS E9* terminator [45]. Recombinant pHEE401 was transformed into *E. coli* DH5α cells, followed by *A. tumefaciens* strain GV3101 via the freeze–thaw method. Construct length and orientation were confirmed by colony PCR and sequencing. Detailed information about the cloning procedure, gRNA module sequences, PCR primers, and colony PCR primers is provided in Supplementary Figure S7 and Table S1.

### Plant transformation and mutation analysis

Four- to six-week-old *A. thaliana* Col-0 wild-type plants were transformed with *A. tumefaciens* as described above. T_0_ seeds were germinated in MS media supplemented with hygromycin (25 mg L^−1^). Antibiotic-resistant transformants were transferred to pots containing soil rite in the growth chamber. To detect mutagenesis at desired sites, genomic DNA was extracted from individual plants of the T_0_ generation using the NucleoSpin Plant II Kit (TaKaRa). Next, target regions were PCR amplified (forward and reverse primers flanked target 1 and target 2, respectively; primer details are provided in Supplementary Table S1) with specific primers and Sanger sequenced. Sequencing data were analyzed in the SnapGene viewer, and editing efficiency was calculated. Additionally, PCR products were resolved on an agarose gel to detect chromosomal fragment deletions. T_0_ plants were selfed to generate T_1_ followed by T_2_ seeds.

### Off-target mutation analysis and identification of ‘transgene-free’ mutants

To analyze off-target effects, Cas-OFFinder (http://www.rgenome.net/cas-offinder/) and CRISPR-P 2.0 (http://crispr.hzau.edu.cn/CRISPR2/) were initially used to predict potential off-target sites for each target or gRNA spacer. Further, fragments of different potential off-target sites were amplified with their corresponding primers (listed in Supplementary Table S1) and sequenced to examine whether any mutation occurred or not.

To identify ‘transgene-free’ T_1_ generation plants, genomic DNA was extracted from T_1_ events using the NucleoSpin Plant II kit (TaKaRa). Subsequently, Cas9-specific primers, gRNA scaffold to downstream vector-specific primers, and *Arabidopsis thaliana* housekeeping gene (*18S rRNA*, NCBI accession number: X16077)-specific primers (listed in Supplementary Table S1) were used to amplify the transgenic elements from the genomic DNA by using the multiplex PCR technique.

### Nematode resistance analysis of mutant plants

Homozygous mutant T_2_ plants were grown in Petri dishes containing MS medium. Two-week-old seedlings were transferred to pots as described above. Three-week-old plants were inoculated with 500 *M. incognita* J2s near the root zone. At 30 days after inoculation, plants were harvested, and different parameters, including numbers of adult females, egg mass, eggs per egg mass, and nematode multiplication factor, were studied. Different phenotypic characters such as plant length, weight, and flowering time were compared between wild-type and mutants to determine whether any growth penalty was incurred by the induced mutation.

### Statistical analysis

The data are presented as the mean ± SE of at least three independent experiments. Data from different bioassays were initially checked for normality using the Shapiro–Wilk test and subjected to a one-way ANOVA test or a *t*-test (for pairwise comparisons between different treatments), followed by the Tukey’s honest significant difference test (for multiple comparisons) in SAS v. 14.1 software.

### Supplementary Information

**Additional file 1: Figure S1. **Amino acid sequence alignment of *A. thaliana* HIPP27 protein (NCBI accession number: NP_201412) with HIPP27 proteins from *Gossypium hirsutum* (XP_040937216), *G. arboreum* (XP_017645826) and *G. raimondii* (XP_012472579). * and **:** indicate identical and similar amino acids, respectively. **Figure S2. **RT-qPCR-based expression analysis of *hipp27* gene in different plant parts and developmental stages of *A. thaliana* Col-0. Fold change in expression was set as 1 in root tissue and 7 days-old-plant, and statistically compared with *hipp27* expression in other plant parts and developmental stages, respectively (no significant difference was observed; Tukey’s HSD test, *P* > 0.05). Gene expression was normalized using two housekeeping genes of *A. thaliana* (ubiquitin and *18S rRNA*). Each bar represents the mean fold change value ± standard error (SE) of qPCR runs in three biological and technical replicates. **Figure S3. **RT-qPCR-based expression analysis of *hipp27* gene and its homologues (*hipp20*, *hipp21*, *hipp22*, *hipp23*, *hipp24*, *hipp25*, *hipp26*) in genome edited line Athipp27-cr-12 and wild-type plants. Fold change in expression of target gene was set as 1 in wild-type, and statistically compared with expression in edited plants. Asterisk indicate significant difference (Tukey’s HSD test, *P* < 0.01) in edited plant compared to the wild-type. Gene expression was normalized using two housekeeping genes of *A. thaliana* (ubiquitin and *18S rRNA*). Each bar represents the mean fold change value ± standard error (SE) of qPCR runs in three biological and technical replicates. **Figure S4. **Comparative phenotyping of *A. thaliana* wild-type and HIPP27 mutant line for different growth parameters. Bars represent mean ± SE. Data were analyzed via Tukey’s HSD test, *P *< 0.05. Bottom panel represents the images of 30-days-old plants in pots containing soil rite. **Figure S5. **RT-qPCR-based expression analysis of defense response genes in shoots (A) and roots (B) of *M. incognita*-infected *A. thaliana* wild-type and HIPP mutants at 2 days after inoculation. Fold change in expression was set as 1 in wild-types and statistically compared with expression in HIPP mutants (no significant difference was observed; Tukey’s HSD test, *P* > 0.05). Gene expression was normalized using two housekeeping genes of *A. thaliana* (ubiquitin and *18S rRNA*). Each bar represents the mean fold change value ± standard error (SE) of qPCR runs in three biological and technical replicates. **Figure S6. **Generation of transgenic Arabidopsis plants. (A) *A. thaliana* Col-0 plants at flowering stage suitable for transformation, (B) Plant transformation with *Agrobactrium* culture via floral dip method, (C) Exposure of plants to low temperature for vernalization, (D) Plants at pod stage for seed collection, (E) Dormancy breaking of T_0_ seeds, (F) seed germination in MS media supplemented with hygromycin antibiotic, (G) Screening of antibiotic resistant seedlings (red circles indicate seeds that did not germinate), (H) Transfer of 14-days-old seedlings (3-4 leaf stage) to pots containing soil rite, (I) Flowering initiated in 30-days-old plants, (J) Plants ready for genotyping and phenotyping analysis. **Figure S7. **Schematic representation of CRISPR/Cas9 vector construction. Amplification with the plasmid pCBC as a template was used to generate a PCR product containing the two targets (flanked by *Bsa* I endonuclease sites), gRNA scaffold, *Arabidopsis* U6 gene terminator (U6-26t) and promoter (U6-29p). PCR forward (F) and reverse (R) primers contained the two target sequences and *Bsa* I site. Two gRNA expression cassettes were assembled into the T-DNA portion of Cas9-expressing binary vector pHEE401 via Golden Gate cloning by replacing the spectinomycin resistance (SpecR) gene. In recombinant pHEE401 (pHEE401:Athipp27-cr), first gRNA expression cassette is driven by the *Arabidopsis* U6 promoter U6-26p. Codon optimized Cas9 expression is driven by an egg cell-specific promoter (EC1p) and *rbcS E9* terminator (rbcS-E9t). NLS, nuclear localization signal; 35Sp, CaMV35S promoter; HygR, Hygromycin resistance; PolyA, CaMV35S terminator; LB, left border; RB, right border. Bottom panel depicts the nucleotide sequence of two sgRNA expression cassette generated in the current study. Gel photograph indicates PCR amplification of expected 846 bp fragment from different colonies of *Agrobacterium tumefaciens* GV3101 harboring the pHEE401:Athipp27-cr construct. M, 100 bp DNA ladder. **Figure S8. **Full-length gel image used in Figs. 4C and 5B, C. No overexposure was used.

**Additional file 2: Table S1.** List of primers used for CRISPR/Cas9 experiment. Annealing temperature – 60ºC. Underlined sequences are BsaI recognition site. Smaller case letters are gRNA spacer or target sequences.

## Data Availability

The datasets generated and/or analysed during the current study are available in the NCBI Genbank repository (https://www.ncbi.nlm.nih.gov/genbank/) and EnsemblPlants repository (https://plants.ensembl.org/Arabidopsis_thaliana/Info/Index). Accession numbers for the datasets analyzed in the current study include AT5G66110, AT1G01490, AT1G06330, AT1G22990, AT1G23000, AT1G29000, AT1G29100, AT1G55790, AT1G56210, AT1G57780, AT1G63950, AT1G71050, AT2G18196, AT2G28090, AT2G36950, AT3G02960, AT3G04900, AT3G05220, AT3G05920, AT3G06130, AT3G07600, AT3G20180, AT3G21490, AT3G48970, AT3G56891, AT4G08570, AT4G10465, AT4G13380, AT4G35060, AT4G38580, AT4G39700, AT5G03380, AT5G17450, AT5G19090, AT5G24580, AT5G26690, AT5G27690, AT5G48290, AT5G52720, AT5G52730, AT5G52740, AT5G52750, AT5G52760, AT5G60800, AT5G63530, NM_126009, XM_021016163, XM_010446534, XM_018628277, XM_013771781, XM_013819665, XM_009152609, XM_023148170, XM_008442628, XM_012617125, XM_039141661, XM_022277399, XM_017790337, XM_020358924, XM_024302046, KF033340, XM_028045898, XM_019310248, XM_014644217.

## Abbreviations

S gene: Susceptibility gene
R gene: Resistance gene
RNAi: RNA interference
RT-qPCR: Reverse transcription Quantitative polymerase chain reaction
NCBI: National center for biotechnology information
aa: amino acids
bp: base pair
CRISPR/Cas9: Clustered regularly interspersed short palindromic repeats/CRISPR associated
MMEJ: Microhomology-mediated end joining
NHEJ: Non-homologous end joining
HDR: Homology directed repair
PAM: Protospacer adjacent motif
crRNA: CRISPR RNA
tracrRNA: transactivating CRISPR RNA
sgRNA: single guide RNA
MF: Multiplication factor
