# Supplementary material for: Functional analysis of a susceptibility gene (HIPP27) in the Arabidopsis thaliana-Meloidogyne incognita pathosystem by using a genome editing strategy
Source: BMC Plant Biol. 2023 Aug 11;23:390. doi: 10.1186/s12870-023-04401-w (PMC10416466; doi:10.1186/s12870-023-04401-w)
Supplement: Supplementary file 1 — Additional file 1: Figure S1. Amino acid sequence alignment of A. thaliana HIPP27 protein (NCBI accession number: NP_201412) with HIPP27 proteins from Gossypium hirsutum (XP_040937216), G. arboreum (XP_017645826) and G. raimondii (XP_012472579). * and : indicate identical and similar amino acids, respectively. Figure S2. RT-qPCR-based expression analysis of hipp27 gene in different plant parts and developmental stages of A. thaliana Col-0. Fold change in expression was set as 1 in root tissue and 7 days-old-plant, and statistically compared with hipp27 expression in other plant parts and developmental stages, respectively (no significant difference was observed; Tukey’s HSD test, P > 0.05). Gene expression was normalized using two housekeeping genes of A. thaliana (ubiquitin and 18S rRNA). Each bar represents the mean fold change value ± standard error (SE) of qPCR runs in three biological and technical replicates. Figure S3. RT-qPCR-based expression analysis of hipp27 gene and its homologues (hipp20, hipp21, hipp22, hipp23, hipp24, hipp25, hipp26) in genome edited line Athipp27-cr-12 and wild-type plants. Fold change in expression of target gene was set as 1 in wild-type, and statistically compared with expression in edited plants. Asterisk indicate significant difference (Tukey’s HSD test, P < 0.01) in edited plant compared to the wild-type. Gene expression was normalized using two housekeeping genes of A. thaliana (ubiquitin and 18S rRNA). Each bar represents the mean fold change value ± standard error (SE) of qPCR runs in three biological and technical replicates. Figure S4. Comparative phenotyping of A. thaliana wild-type and HIPP27 mutant line for different growth parameters. Bars represent mean ± SE. Data were analyzed via Tukey’s HSD test, P < 0.05. Bottom panel represents the images of 30-days-old plants in pots containing soil rite. Figure S5. RT-qPCR-based expression analysis of defense response genes in shoots (A) and roots (B) of M. incognita-infected A [file 12870_2023_4401_MOESM1_ESM.pdf]

Figure S1. Amino acid sequence alignment of *A. thaliana* HIPP27 protein (NCBI accession number: NP_201412) with HIPP27 proteins from *Gossypium hirsutum* (XP_040937216), *G. arboreum* (XP_017645826) and *G. raimondii* (XP_012472579). * and **:** indicate identical and similar amino acids, respectively.

```
A.thaliana      MGFRDICY-------RKHH-KKLKQFQKVEIKVKMDCEGCERRVRKSVEGMKGVSKVTVD   52
G.raimondii     MGFLDSVFEFFDCDWPSHKKLKKKILQTVEIKVKMDCEGCERKVKKSVQGMKGVTQVEVN   60
G.hirsutum      MGFLDSVFEFFDCDWPSHKKLKKKPLRTVEIKVKLDCEGCERKVKKSVQGMKGVTQVEVN   60
G.arboreum      MGFLDSVFEFFDCDWPSHKKLKKKPLQTVEIKVKMDCEGCERKVKKSVQGMKGVTQVEVN   60
                *** *  :        .*:  * * ::.******:*******:*:***:*****::* *:

A.thaliana      PKQSKLTVEGFVQPSKVVHRVMHRTGKKAELWPYVPYEVVPHPYAPGAYDKKAPPGYVRN   112
G.raimondii     PKQSKLTVVGYVDPDKVLERVRHRTGKKVEFWPYVPYDLVPHPYAPGAYDKKAPPGYVRN   120
G.hirsutum      PKQSKLTVVGYVDPDKVLDRVRHRTGKKVEFWPYVPYDVVPHPYAPGAYDKKAPPGYVRN   120
G.arboreum      PKQSKLTVVGYVDPDKVLDRVRHRTGKKVEFWPYVPYDVVPHPYAPGAYDKKAPPGYVRN   120
                ******** *:*:*.**:.** ******.*:******::*********************

A.thaliana      ALADPLVAPLARASSFEVKYTSAFSDDNPNACTIM   147
G.raimondii     VVGDPQAGELARATSFEVKYTTAFSDENPNACVIM   155
G.hirsutum      VVGDPQAGELARATSFEVKYTTAFSDENPNACVII   155
G.arboreum      VVGDPQAGELARATSFEVKYTTAFSDENPNACVIM   155
                .:.** .. ****:*******:****:*****.*:
```

Figure S2. RT-qPCR-based expression analysis of *hipp27* gene in different plant parts and developmental stages of *A. thaliana* Col-0. Fold change in expression was set as 1 in root tissue and 7 days-old-plant, and statistically compared with *hipp27* expression in other plant parts and developmental stages, respectively (no significant difference was observed; Tukey's HSD test, $P > 0.05$). Gene expression was normalized using two housekeeping genes of *A. thaliana* (ubiquitin and *18S rRNA*). Each bar represents the mean fold change value ± standard error (SE) of qPCR runs in three biological and technical replicates.

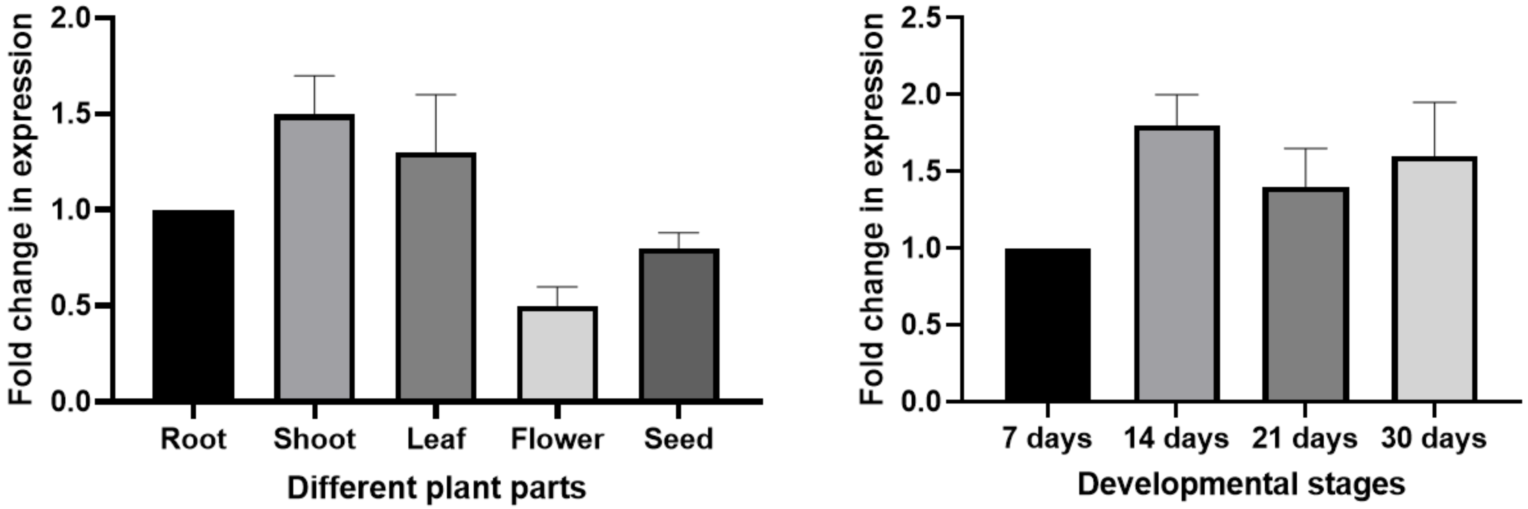

Figure S3. RT-qPCR-based expression analysis of *hipp27* gene and its homologues (*hipp20*, *hipp21*, *hipp22*, *hipp23*, *hipp24*, *hipp25*, *hipp26*) in genome edited line Athipp27-cr-12 and wild-type plants. Fold change in expression of target gene was set as 1 in wild-type, and statistically compared with expression in edited plants. Asterisk indicate significant difference (Tukey's HSD test, $P < 0.01$) in edited plant compared to the wild-type. Gene expression was normalized using two housekeeping genes of *A. thaliana* (ubiquitin and *18S rRNA*). Each bar represents the mean fold change value ± standard error (SE) of qPCR runs in three biological and technical replicates.

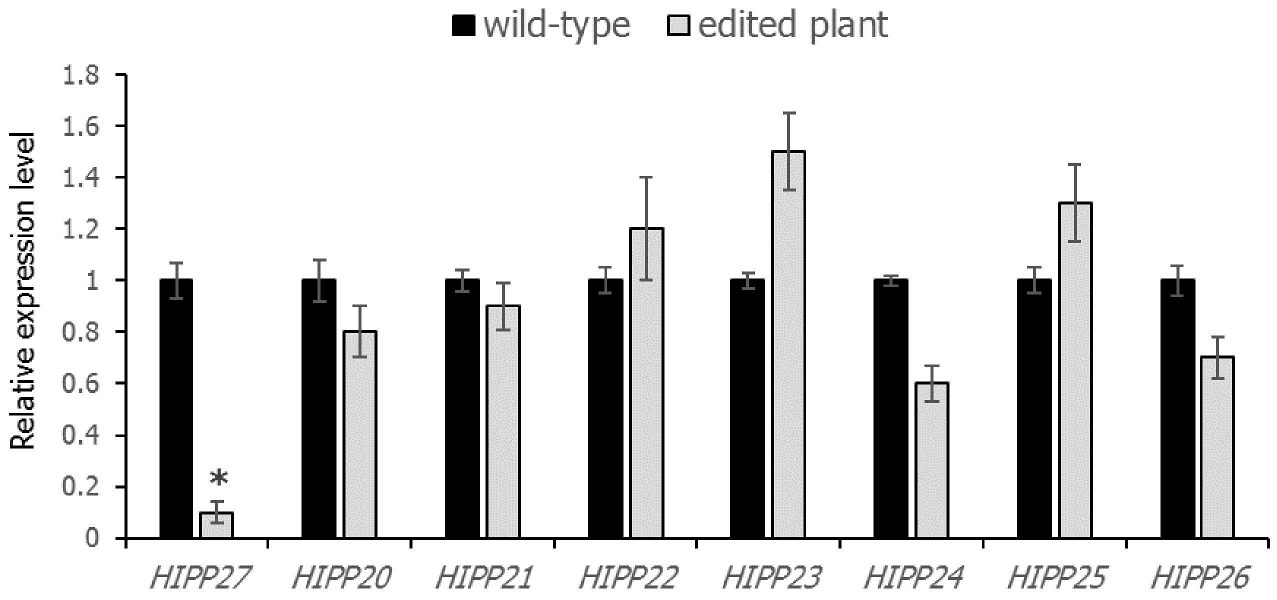

Figure S4. Comparative phenotyping of *A. thaliana* wild-type and HIPP27 mutant line for different growth parameters. Bars represent mean ± SE. Data were analyzed via Tukey's HSD test, $P < 0.05$. Bottom panel represents the images of 30-days-old plants in pots containing soil rite.

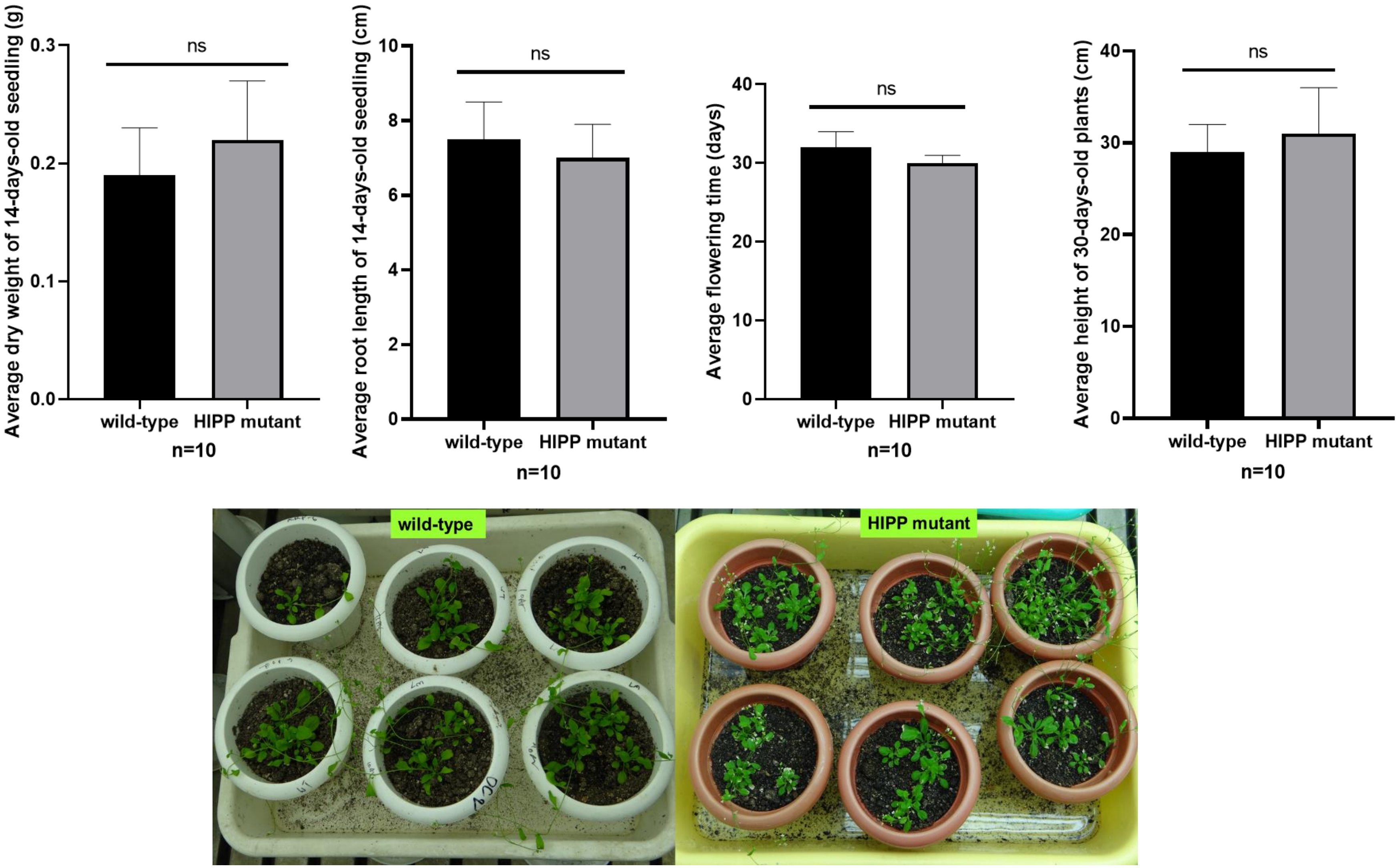

Figure S5. RT-qPCR-based expression analysis of defense response genes in shoots **(A)** and roots **(B)** of *M. incognita*-infected *A. thaliana* wild-type and HIPP mutants at 2 days after inoculation. Fold change in expression was set as 1 in wild-types and statistically compared with expression in HIPP mutants (no significant difference was observed; Tukey's HSD test, $P > 0.05$). Gene expression was normalized using two housekeeping genes of *A. thaliana* (ubiquitin and *18S rRNA*). Each bar represents the mean fold change value ± standard error (SE) of qPCR runs in three biological and technical replicates.

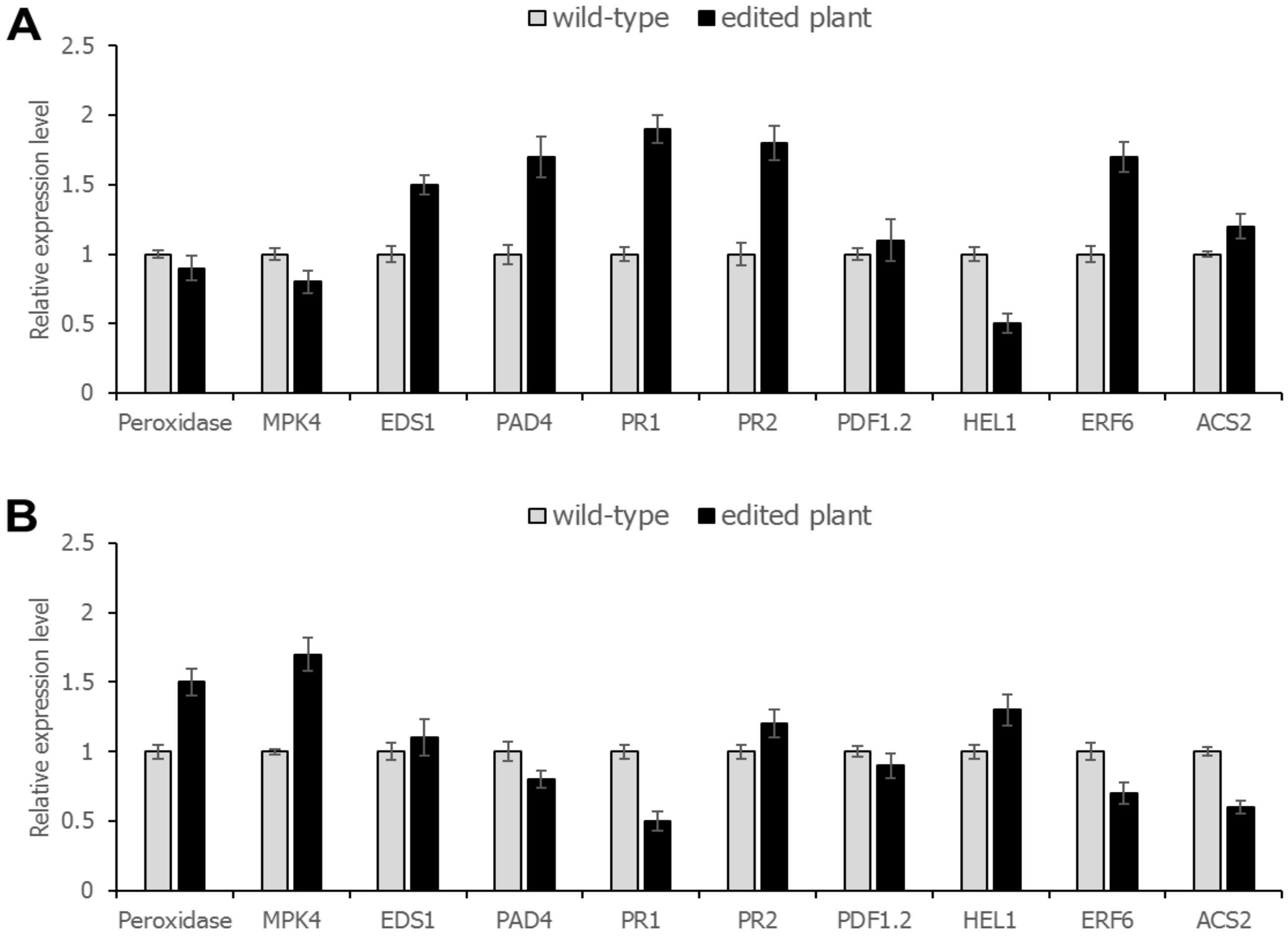

Figure S6. Generation of transgenic *Arabidopsis* plants. **(A)** *A. thaliana* Col-0 plants at flowering stage suitable for transformation, **(B)** Plant transformation with *Agrobactrium* culture via floral dip method, **(C)** Exposure of plants to low temperature for vernalization, **(D)** Plants at pod stage for seed collection, **(E)** Dormancy breaking of T_0 seeds, **(F)** seed germination in MS media supplemented with hygromycin antibiotic, **(G)** Screening of antibiotic resistant seedlings (red circles indicate seeds that did not germinate), **(H)** Transfer of 14-days-old seedlings (3-4 leaf stage) to pots containing soil rite, **(I)** Flowering initiated in 30-days-old plants, **(J)** Plants ready for genotyping and phenotyping analysis.

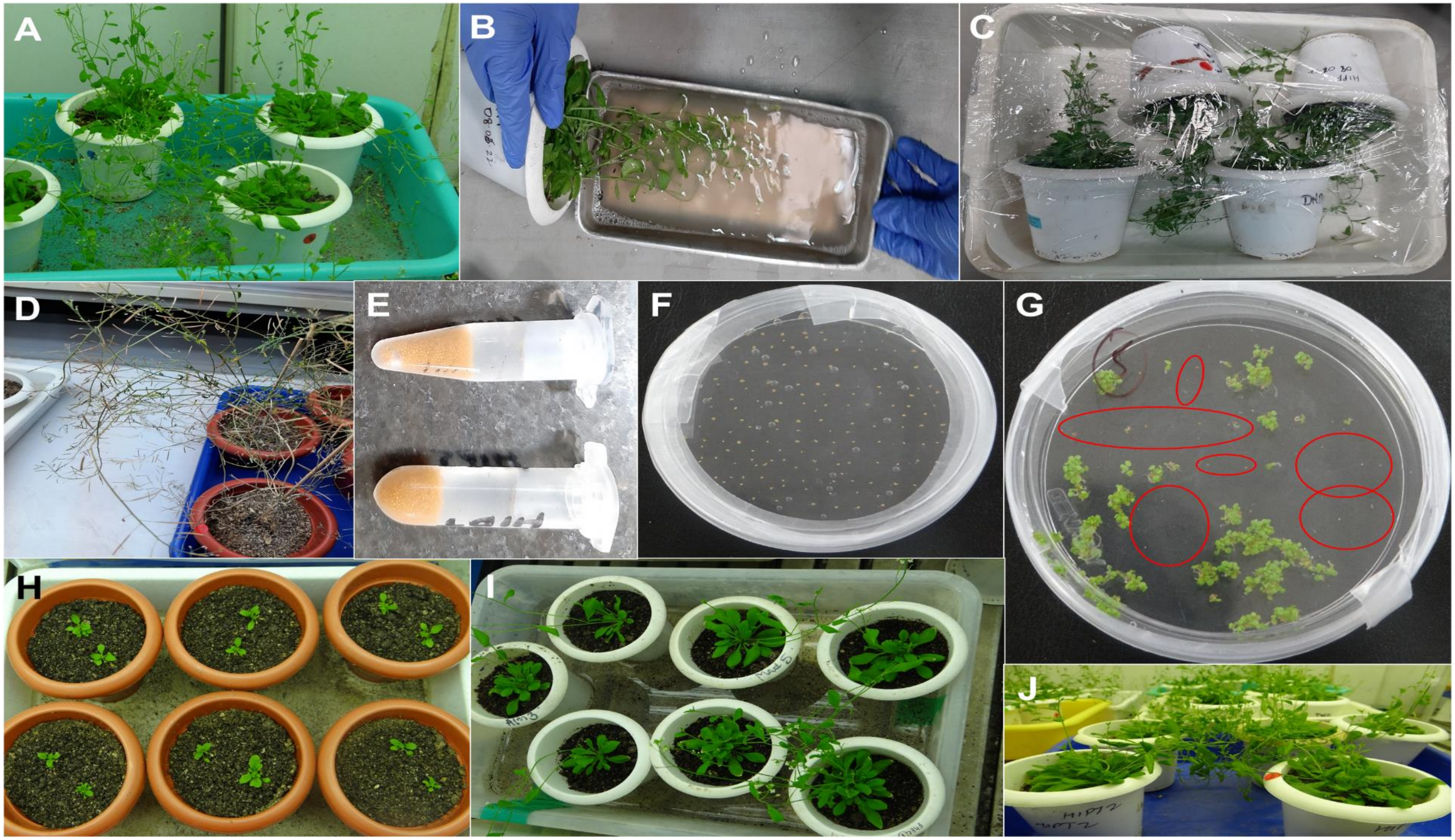

Figure S7. Schematic representation of CRISPR/Cas9 vector construction. Amplification with the plasmid pCBC as a template was used to generate a PCR product containing the two targets (flanked by *Bsa* I endonuclease sites), gRNA scaffold, *Arabidopsis* U6 gene terminator (U6-26t) and promoter (U6-29p). PCR forward (F) and reverse (R) primers contained the two target sequences and *Bsa* I site. Two gRNA expression cassettes were assembled into the T-DNA portion of Cas9-expressing binary vector pHEE401 via Golden Gate cloning by replacing the spectinomycin resistance (SpecR) gene. In recombinant pHEE401 (pHEE401:Athipp27-cr), first gRNA expression cassette is driven by the *Arabidopsis* U6 promoter U6-26p. Codon optimized Cas9 expression is driven by an egg cell-specific promoter (EC1p) and *rbcS E9* terminator (rbcS-E9t). NLS, nuclear localization signal; 35Sp, CaMV35S promoter; HygR, Hygromycin resistance; PolyA, CaMV35S terminator; LB, left border; RB, right border. Bottom panel depicts the nucleotide sequence of two sgRNA expression cassette generated in the current study. Gel photograph indicates PCR amplification of expected 846 bp fragment from different colonies of *Agrobacterium tumefaciens* GV3101 harboring the pHEE401:Athipp27-cr construct. M, 100 bp DNA ladder.

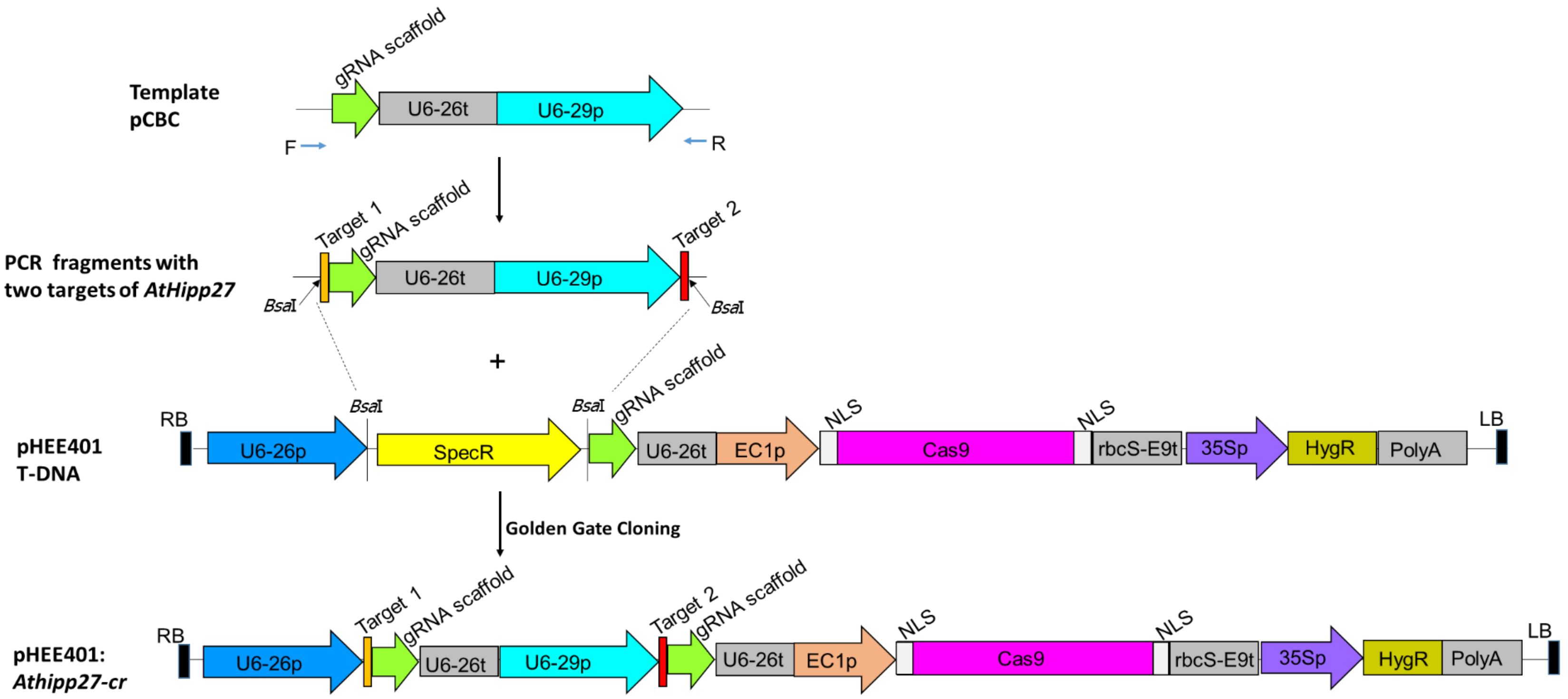

(U6-26p)-(Target-1)-(sgRNA-scaffold)-(U6-26t)-(U6-29p)-(Target-2)-(sgRNA-scaffold)-(U6-26t)

CGACTTGCCTTCCGCACAATACATCATTTCTTCTTAGCTTTTTTTCTTCTTCTTCGTTCATACAGTTTTTTTTTGTTTATCAGCTTACATTTTCTTGAA
CCGTAGCTTTCGTTTTCTTCTTTTTAACTTTCCATTCGGAGTTTTTGTATCTTGTTTCATAGTT**TGTCCCAGGATTAGAATGATTAGGC**ATCGAAC
CTTCAAGAATTTGATTGAATAAAACATCTTCATTCTTAAGATATGAAGATAATCTTCAAAAGGCCCCTGGGAATCTGAAAGAAGAGAAGCAGGC
CCATTTATATGGGAAAGAACAATAGTATTTCTTATATAGGCCCATTTAAGTTGAAAACAATCTTCAAAAGTCCCACATCGCTTAGATAAGAAAAC
GAAGCTGAGTTTATATACAGCTAGAGTCGAAGTAGTGATTGACTGCGAAGGGTGCGAGAGAGTTTTAGAGCTAGAAATAGCAAGTTAAAATAA
GGCTAGTCCGTTATCAACTTGAAAAAGTGGCACCGAGTCGGTGCTTTTTTTTGCAAAATTTTCCAGATCGATTTCTTCTTCCTCTGTTCTTCGGCGT
TCAATTTCTGGGGTTTTCTCTTCGTTTTCTGTAACTGAAACCTAAAATTTGACCTAAAAAAAATCTCAAATAATATGATTCAGTGGTTTTGTACTTT
TCAGTTAGTTGAGTTTTGCAGTTCCGATGAGATAAACCAATATTAATCCAAACTACTGCAGCCTGACAGACAAATGAGGATGCAAACAATTTTAA
AGTTTATCTAACGCTAGCTGTTTTGTTTCTTCTCTCTGGTGCACCAACGACGGCGTTTTCTCAATCATAAAGAGGCTTGTTTTACTTAAGGCCATA
ATGTTGATGGATCGAAAGAAGAGGGCTTTTAATAAACGAGCCCGTTTAAGCTGTAAACGATGTCAAAAACATCCCACATCGTTCAGTTGAAAAT
AGAAGCTCTGTTTATATATTGGTAGAGTC**GCTAAGAGATTGGCATCGGACAGG**GAAGAAGGGTTTTAGAGCTAGAAATAGCAAGTTAAAATA
AGGCTAGTCCGTTATCAACTTGAAAAAGTGGCACCGAGTCGGTGCTTTTTTTTGCAAAATTTTCCAGATCGATTTCTTCTTCCTCTGTTCTTCGGC
GTTCAATTTCTGGGGTTTTCTCTTCGTTTTCTGTAACTGAAACCTAAAATTTGACCTAAAAAAAATCTCAAATAATATGATTCAGTGGTTTTGTAC
TTTTCAGTTAGTTGAGTTTTGCAGTTCCGATGAGATAAACCAATA

Bold letters represent primer-binding sequences for colony PCR.

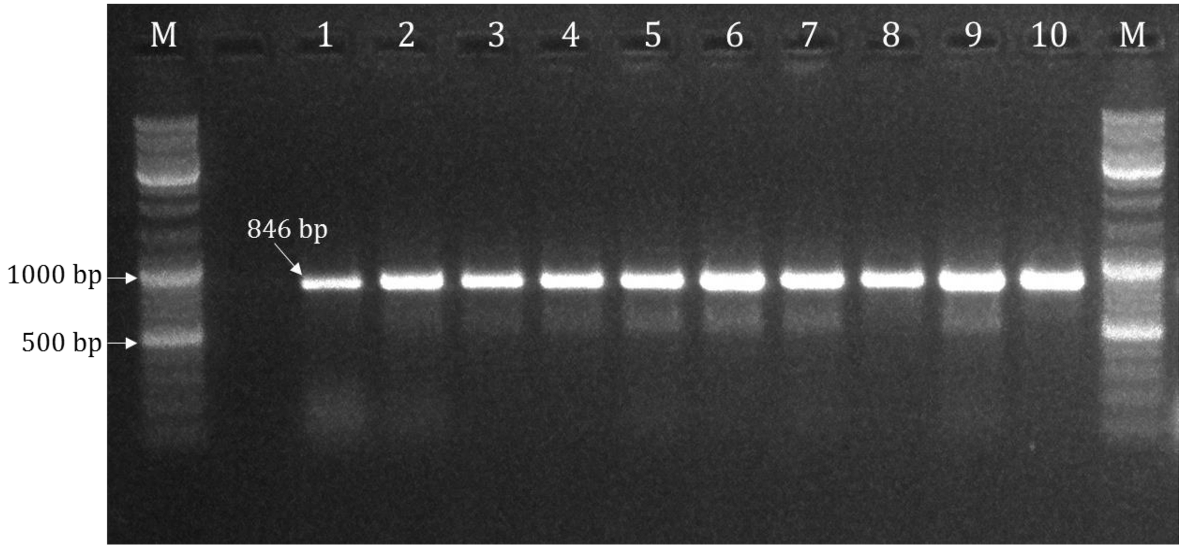

Figure S8. Full-length gel image used in Figure 4C, Figure 5B, 5C. No overexposure was used.

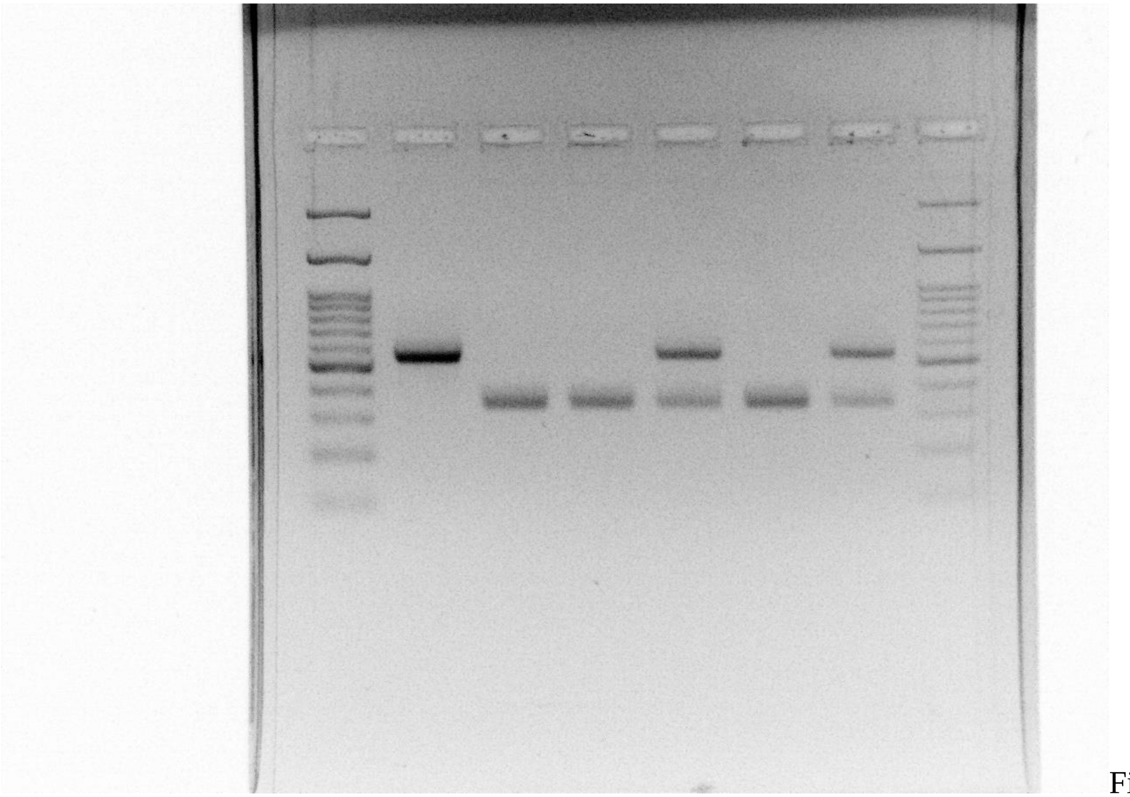

Figure 4C

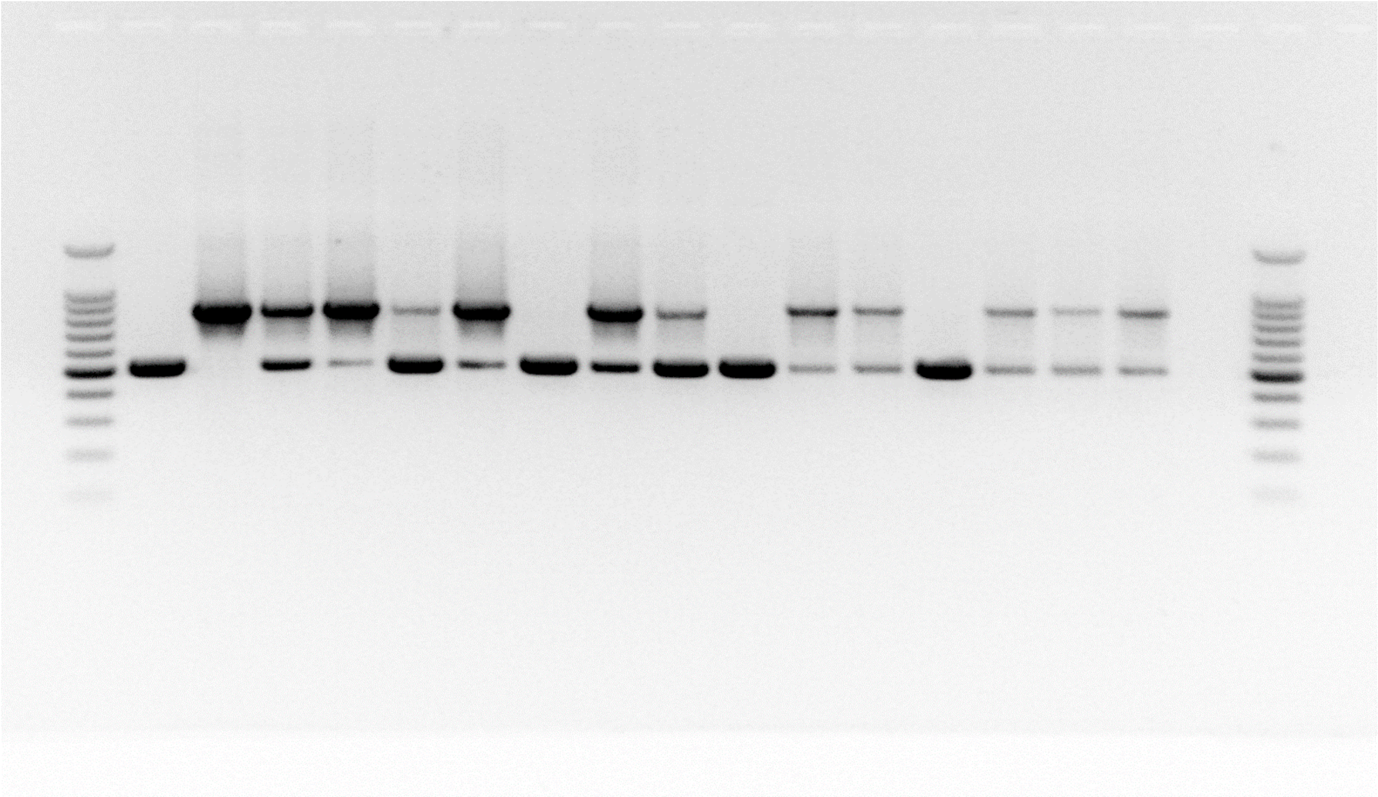

Figure 5A

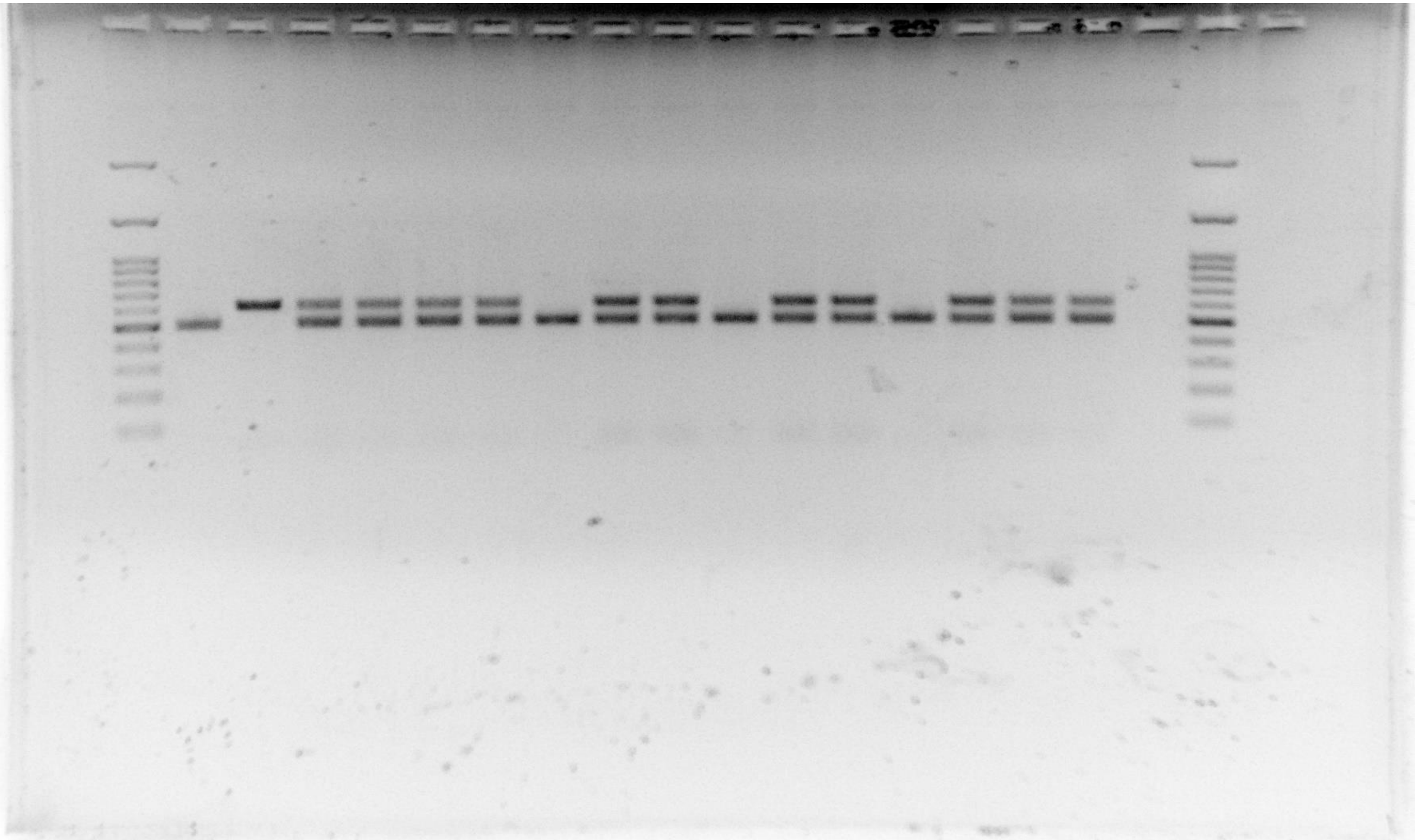

Figure 5B
