# Supplementary material for: Functional analysis of a susceptibility gene (HIPP27) in the Arabidopsis thaliana-Meloidogyne incognita pathosystem by using a genome editing strategy
Source: BMC Plant Biol. 2023 Aug 11;23:390. doi: 10.1186/s12870-023-04401-w (PMC10416466; doi:10.1186/s12870-023-04401-w)
Supplement: Supplementary file 2 — Additional file 2: Table S1. List of primers used for CRISPR/Cas9 experiment. Annealing temperature – 60ºC. Underlined sequences are BsaI recognition site. Smaller case letters are gRNA spacer or target sequences. [file 12870_2023_4401_MOESM2_ESM.pdf]

Table S1. List of primers used for CRISPR/Cas9 experiment. Annealing temperature – 60ºC. Underlined sequences are *Bsa*I recognition site. Smaller case letters are gRNA spacer or target sequences.

**Primer name**	**Sequence (5´- 3´)**	**Purpose**
pCXSN:hipp_F	ATGGGCTTCCGAGACATTTGT	Overexpression of *Athipp27* gene
pCXSN:hipp_R	TCACATGATGGTACAAGCGTTG	
pCXGUS-P:hipp_F	AGGAGAGTAAGTGGTTTTCCATC	Overexpression of Athipp27 promoter: GUS
pCXGUS-P:hipp_R	CCTTCTCTGGTAACTTCAAATTG	
qPCR-HIPP27_F	ATGCATCGGACAGGGAAGAA	qPCR analysis of *Athipp27* gene
qPCR-HIPP27_R	AAGCGTTGGGATTGTCATCG	
qPCR-UBQ_F	GGAAAGACCATCACCCTTGA	*A. thalina* Ubiquitin gene as internal control in qPCR
qPCR-UBQ_R	ATCCTCAAGCTGCTTTCCAG	
qPCR-18S_F	GGTGGTAACGGGTGACGGAGAAT	*A. thalina 18S rRNA* gene as internal control in qPCR
qPCR-18S_R	CGCCGACCGAAGGGACAAGCCGA	
Target1_Bsa_F	ATATATGGTCTCGATTGactgcgaagggtgcgagagaGTT	gRNA1 and gRNA2 cassette construction for CRISPR/Cas9 experiment
Target1_gRNA scaffold_F	TGactgcgaagggtgcgagagaGTTTTAGAGCTAGAAATAGC	
Target2_U6-29p_R	AACccttcttccctgtccgatgcCAATCTCTTAGTCGACTCTAC	
Target2_Bsa_R	ATTATTGGTCTCGAAACccttcttccctgtccgatgcCAA	
U6-26p_F	TGTCCCAGGATTAGAATGATTAGGC	Colony PCR and sequencing of the construct
U6-29p-Target2_R	GCTAAGAGATTGgcatcggacagg	
Cas9_F	TCACTAAGCACGTTGCGCAG	Detection of Cas9 protein encoding gene in mutants
Cas9_R	CGCTTCCTGCCGTTCTCC	
At_Hipp_F	CGTAATCGGCGCTAATGGG	Detection of *Athipp27* gene in mutants
At_Hipp_R	AAGCGTTGGGATTGTCATCG	
gRNA scaffold_F	GTTTTAGAGCTAGAAATAGCAAGT	Detection of gRNA scaffold to downstream vector in mutants
pHEE401_R	CTCAAGTCTCCTAGTTTCTCTGT	
At_18S_F	ATTAACAGGGACAGTCGGGG	Detection of *18S rRNA* gene in

At_18S_R	GATGCCTCCACGTAGCTAGT	mutants
AT1G16270_F	CCGTCAGGTGCCAATCCATA	Off-target mutation analysis
AT1G16270_R	TCATCTGAGGATCCACACGG	
AT1G60920_F	TCTCTCCGGGGACTCAAATC	
AT1G60920_R	GCCTCTTTCAACTCGTCGAC	
AT4G36648_F	TGAGAGGTTGGTGGTTCGAA	
AT4G36648_R	TCCATGTTTAGACGCCGTTG	
Peroxidase_F	ACGTGGAATTGGGAAGGCTA	qPCR analysis of defense response genes
Peroxidase_R	TGCGCGAATCCTAATGTGTG	
MPK4_F	TAAGCCCAGCGTAACAGTGA	
MPK4_R	ACGTCTTAGAGATCAGCGGG	
EDS1_F	TACCTTGAGCCTCGTTGTGT	
EDS1_R	GGGCAAGAACATGAGGCAAA	
PAD4_F	TCCTCTGCTCGGAAACCAAT	
PAD4_R	GAGTTGCTGTGGTGTTGAGG	
PR1_F	TGCTCTTGTTCTTCCCTCGA	
PR1_R	CTAACCCACATGTTCACGGC	
PR2_F	TCCGGTACATCAACGTTGGA	
PR2_R	AAGGGAGATTGCTTGCTTGC	
PDF1.2_F	TCTCTTTGCTGCTTTCGACG	
PDF1.2_R	ACTTGTGTGCTGGGAAGACA	
HEL1_F	CACGTGGGATGCTGATAAGC	
HEL1_R	CATCCAAATCCAAGCCTCCG	
ERF6_F	CCTACTACTGCCACCACCAA	
ERF6_R	ACAGTAACGCGAGGAGGATT	

ACS2_F	ATGTGTCTCCTGGCTCTTCC	
ACS2_R	GTCTGCGTCCATTTCAGCTT	
qPCR-HIPP20_F	AAGGAGGAAGCGGAAAGTCA	qPCR analysis of *Athipp20* gene
qPCR-HIPP20_R	TCACCTCCACCGATTTCACA	
qPCR-HIPP21_F	GACACGTGGACCCAAACAAG	qPCR analysis of *Athipp21* gene
qPCR-HIPP21_R	GATTACGGATGTGGCCTGC	
qPCR-HIPP22_F	CCGGAGCTTACGACAAGAGA	qPCR analysis of *Athipp22* gene
qPCR-HIPP22_R	CGTTAGGGTTCTCGTCGCTA	
qPCR-HIPP23_F	ACAGTGAGTGGTTATGCGGA	qPCR analysis of *Athipp23* gene
qPCR-HIPP23_R	CCAGGCGGTGCTTTCTTATC	
qPCR-HIPP24_F	CAGGCGTATGACAAGAAGGC	qPCR analysis of *Athipp24* gene
qPCR-HIPP24_R	AGGGTTCTCGTCGCTAAACA	
qPCR-HIPP25_F	TGGCTCGTATCATTCACCGA	qPCR analysis of *Athipp25* gene
qPCR-HIPP25_R	GTCGGGGCTCTGTTATCGTA	
qPCR-HIPP26_F	GTCTTCTGTGACGTTGGAGC	qPCR analysis of *Athipp26* gene
qPCR-HIPP26_R	CAGAGCTCGACTTTCTTGCC	
